# High-throughput screening of catalytically active inclusion bodies using laboratory automation and Bayesian optimization

**DOI:** 10.1186/s12934-024-02319-y

**Published:** 2024-02-24

**Authors:** Laura Marie Helleckes, Kira Küsters, Christian Wagner, Rebecca Hamel, Ronja Saborowski, Jan Marienhagen, Wolfgang Wiechert, Marco Oldiges

**Affiliations:** 1 Institute of Bio- and Geosciences IBG-1: Biotechnology, Forschungszentrum Jülich GmbH, 52425 Jülich, Germany; 2 Institute of Biotechnology, RWTH Aachen University, 52074 Aachen, Germany; 3 Computational Systems Biotechnology (AVT.CSB), RWTH Aachen University, 52074 Aachen, Germany

**Keywords:** Automation, Bayesian statistical model, Catalytically active inclusion bodies, Enzyme immobilization, Protein engineering, Thompson sampling

## Abstract

**Background:**

In recent years, the production of inclusion bodies that retain substantial catalytic activity was demonstrated. These catalytically active inclusion bodies (CatIBs) are formed by genetic fusion of an aggregation-inducing tag to a gene of interest via short linker polypeptides. The resulting CatIBs are known for their easy and cost-efficient production, recyclability as well as their improved stability. Recent studies have outlined the cooperative effects of linker and aggregation-inducing tag on CatIB activities. However, no a priori prediction is possible so far to indicate the best combination thereof. Consequently, extensive screening is required to find the best performing CatIB variant.

**Results:**

In this work, a semi-automated cloning workflow was implemented and used for fast generation of 63 CatIB variants with glucose dehydrogenase of *Bacillus subtilis* (*Bs*GDH). Furthermore, the variant *Bs*GDH-PT-CBDCell was used to develop, optimize and validate an automated CatIB screening workflow, enhancing the analysis of many CatIB candidates in parallel. Compared to previous studies with CatIBs, important optimization steps include the exclusion of plate position effects in the BioLector by changing the cultivation temperature. For the overall workflow including strain construction, the manual workload could be reduced from 59 to 7 h for 48 variants (88%). After demonstration of high reproducibility with 1.9% relative standard deviation across 42 biological replicates, the workflow was performed in combination with a Bayesian process model and Thompson sampling. While the process model is crucial to derive key performance indicators of CatIBs, Thompson sampling serves as a strategy to balance exploitation and exploration in screening procedures. Our methodology allowed analysis of 63 *Bs*GDH-CatIB variants within only three batch experiments. Because of the high likelihood of TDoT-PT-*Bs*GDH being the best CatIB performer, it was selected in 50 biological replicates during the three screening rounds, much more than other, low-performing variants.

**Conclusions:**

At the current state of knowledge, every new enzyme requires screening for different linker/aggregation-inducing tag combinations. For this purpose, the presented CatIB toolbox facilitates fast and simplified construction and screening procedures. The methodology thus assists in finding the best CatIB producer from large libraries in short time, rendering possible automated Design-Build-Test-Learn cycles to generate structure/function learnings.

**Supplementary Information:**

The online version contains supplementary material available at 10.1186/s12934-024-02319-y.

## Background

Catalytically active inclusion bodies (CatIBs) are inclusion bodies that were demonstrated to retain substantial catalytic activity [1–11]. CatIBs are known for their easy and cost-efficient production, recyclability as well as high stability. Due to their ability to self-aggregate in a carrier-free, biodegradable form, further laborious immobilization steps and additional reagents can be avoided [12]. These characteristics make CatIBs a promising, purely biological alternative to traditional immobilization techniques.

CatIBs can be formed by genetic fusion of an aggregation-inducing tag to a gene of interest via short linker polypeptides and overproduction of the resulting fusion gene in *Escherichia* *coli*. The choice of linker and aggregation-inducing tag strongly influences CatIB activities, as was previously shown for CatIBs of the lysine decarboxylase from *E. coli* or of the glucose dehydrogenase from *Bacillus subtilis* (*Bs*GDH). For both enzymes, the rigid Proline/Threonine (PT) linker led to higher activities compared to the flexible Serine/Glycine (SG) linker [10, 11]. Besides the linker and aggregation-inducing tag, also the terminus of the enzyme, which is used for fusion, influences the activity of CatIBs. While CatIB formation using the N-terminus often failed in previous studies, fusion to the C-terminus was shown to be more successful [13].

Since several factors influence the formation of active CatIBs, a priori prediction of suitable combinations of target enzyme, linker and aggregation-inducing tag is not possible at the current state of knowledge [12, 14]. As a consequence, many different genetic variants need to be constructed and tested, ideally in a Design-Build-Test-Learn (DBTL) cycle that can be automated for enhanced throughput. For the generation of a large CatIB library, i.e., the Build phase of DBTL, a modern cloning technique is essential to speed up the whole process. In previous studies, traditional cloning processes, consisting of restriction digestion, ligation, polymerase chain reaction (PCR) and gel clean-up steps, were applied for the construction of CatIBs [1–9]. However, these cloning workflows are laborious and time-consuming. In comparison, Golden Gate Assembly (GGA) is a fast and simple cloning technique. Since it is easy to automate, GGA can be used to construct many different CatIB variants in parallel [14]. Moreover, GGA was already successfully applied for the construction of ten lysine decarboxylase and 14 glucose dehydrogenase CatIBs [10, 11].

Construction of large libraries also requires high-throughput screening to evaluate the respective strains. Here, automation and miniaturization are essential for screening, which can be realized by automated microbioreactors, i.e., miniaturized, high-throughput cultivation systems embedded into a liquid-handling robotic platform [15]. In order to test variants on such a platform in high-throughput, automation of cultivation, purification of CatIBs, as well as analytical procedures are required.

Finally, a DBTL cycle for CatIBs requires Learn and Design phases, where the high-throughput data is analyzed and used to suggest new experimental designs or candidates. Statistical methods for experimental planning such as Design of Experiments (DoE) are popular in industry and academia, fostering process understanding and reducing the amount of experiments required to identify critical process parameters and connecting them to critical quality attributes [16–18]. As an alternative to classical DoE, Bayesian optimization gained popularity over the past decade, enhancing process optimization and experimental design in various fields [19–21], including bioprocess development [22, 23]. Due to the iterative nature of DBTL, sequential optimization of results after each round of experiments is required. At the same time, batch-wise suggestions are necessary to match the high-throughput setup of microbioreactors. In this context, a previous study successfully demonstrated how a Bayesian process model and Thompson sampling, an algorithm to sample from probability distributions of key performance indicators (KPIs), could be combined for iterative screening of a PETase strain library [24]. For further background information on Bayesian statistics and Thompson sampling, we refer the interested reader to available tutorials and textbooks, e.g., [25–27].

In this study, we pave the way for a CatIB DBTL cycle by combining workflow automation with process modeling. In a first step, we introduce a semi-automated cloning procedure for parallel construction of CatIB variants. Subsequently, we demonstrate an automated screening workflow to test 63 generated CatIB variants of *Bs*GDH on our automated liquid handling platform. The enzyme was chosen due to its relevance as a cofactor regeneration module, catalyzing the reaction of β-D-glucose to D-glucono-1,5-lactone with NAD(P)^+^ as a cofactor [28]. Finally, a Bayesian process model for CatIB screening is applied to model the reaction rate of different variants in a high-throughput assay. Combined with Thompson sampling for iterative experiment planning, we conduct a screening to identify the best CatIB variant, which could be transferred to large-scale bioreactor experiments for further optimization.

## Results and discussion

### Semi-automated strain construction to generate 63 different *Bs*GDH-CatIBs

**Fig. 1:**
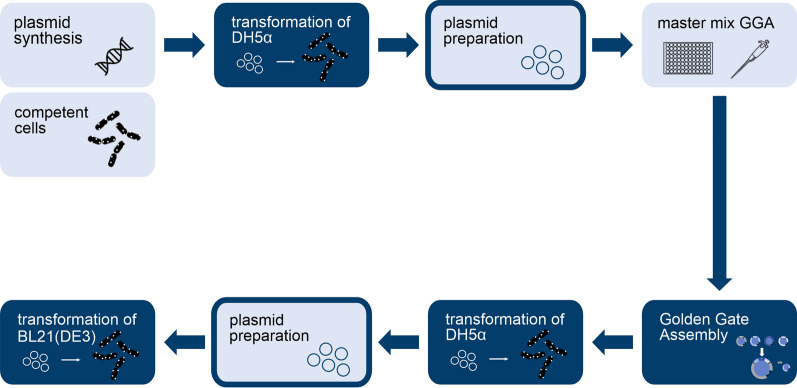
Semi-automated CatIB construction workflow. Plasmids were commercially synthesized by Synbio Technologies (Monmouth Junction, US). Manual (light blue boxes), accelerated (dark blue framed boxes) and automated (dark blue boxes) cloning steps were performed to construct *E.* *coli* BL21(DE3) strains containing different episomally encoded CatIB variants

As a first step towards DBTL for CatIBs, the construction of genetic variants was partially automated to enhance throughput. More precisely, construction of up to 96 CatIB variants in parallel was targeted (Fig. 1).

A special focus was set to repetitive and laborious steps, while steps that are only performed a few times or at large scales were still conducted manually. Most importantly, transformation and GGA were fully automated using the Opentrons system, while plasmid preparation was accelerated by parallelization. A detailed description of the semi-automated workflow is given in Additional file 1. The optimized semi-automated CatIB cloning process in combination with GGA presents a fast and efficient tool to construct CatIB variants in parallel. For the generation of 96 CatIB variants only 11 h of manual work are needed, which corresponds to 17% of the worktime for a traditional cloning workflow.

For the case study with *Bs*GDH, it was applied to generate 63 CatIB variants. SG or PT linker were combined with one of eight aggregation-inducing tags (TDoT, 18AWT, L6KD, GFIL8, 3HAMP, CBDCell, TorA and ELK16), which were fused to the C- and N-terminus of *Bs*GDH [2, 3, 6–9, 13, 29]. In addition to these variants, studies with varied linkers and aggregation-inducing tags were performed, where the influence of different lengths of serine/proline linkers (G1, G2, G3, G4, G5, G10 or P1, P2, P3, P4, P5, P10) as well as of the L6KD tag (L12KD, L24KD, L36KD, L48KD, L96KD, (L6KD)_2_, (L6KD)_4_, (L6KD)_6_, (L6KD)_8_, (L6KD)_16_) were tested for CatIB formation. In both linker and aggregation-inducing tag studies, the influence of fusion to the N- and C-terminus was tested as an additional factor. The implemented semi-automated cloning workflow was used to construct the variants as well as a plasmid encoding the soluble *Bs*GDH_WT_. The genetic sequences of each expression plasmid were verified via sequencing. 13 CatIB variants could not be successfully cloned within three attempts, potentially due to a non-viable phenotype. These variants were not considered for CatIB screening (Additional file 1: Figure S1). However, 63 out of 76 CatIB constructs (83%) were successfully constructed with the semi-automated workflow and were analyzed in more detail via CatIB screening.

### Overview of the CatIB screening workflow

**Fig. 2:**
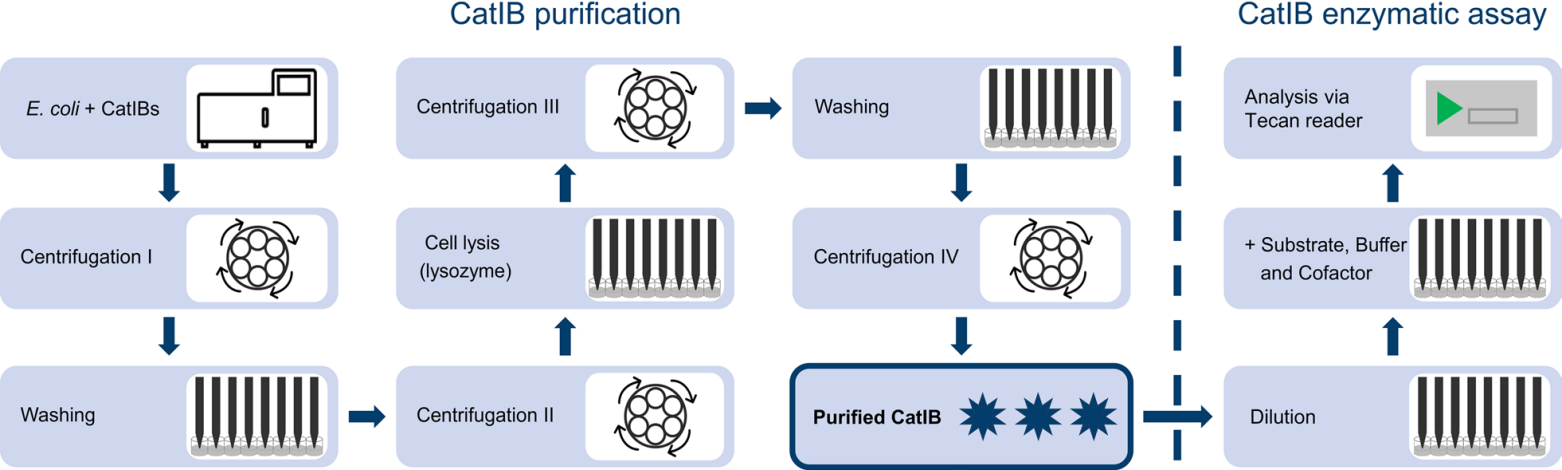
Overview of the CatIB purification (left) and screening workflow (right) with Tecan Freedom Evo®200 platform. Purified CatIBs (highlighted box) indicate the transitional step from purification to enzymatic assay

For screening of large CatIB libraries, which corresponds to the Test phase of DBTL, the automation of the whole workflow was essential. At the beginning of the workflow, parallelized cultivation of *E. coli* BL21(DE3) strains with various CatIB combinations was performed. This step was realized using a BioLector I with a FlowerPlate as cultivation system (Fig. 2).

The CatIB purification process consisted of several resuspension, centrifugation and washing steps. The first centrifugation step ensured the separation of the cells from the cultivation media. After washing and a second centrifugation step, the cells were lysed via resuspension of the pellet in BugBuster® with additional lysozyme. The third centrifugation step was performed to separate the soluble from the insoluble cell fraction. The supernatant containing the soluble enzyme fraction was removed from the pellet fraction. The second washing step with Milli-Q® water was performed to wash the insoluble fraction that contained the CatIBs. The washed CatIB fraction was obtained after a last centrifugation step to remove the Milli-Q®. The CatIB pellets were resuspended and diluted 1:20 with Milli-Q® water. Pre-warming of the CatIB/water suspension and the assay solution was performed at 40 °C for 10 min to avoid temperature effects in the fluorescence-based assay. After combining these two solutions, the mixture was incubated and analyzed at 37 °C in a photometer to enable online measurement of the NADH formation. This process, which is further described in “Automated protein production and protein purification” section, was used for the subsequent analyses.

### Optimization of the CatIB workflow – BioLector positional bias

**Fig. 3:**
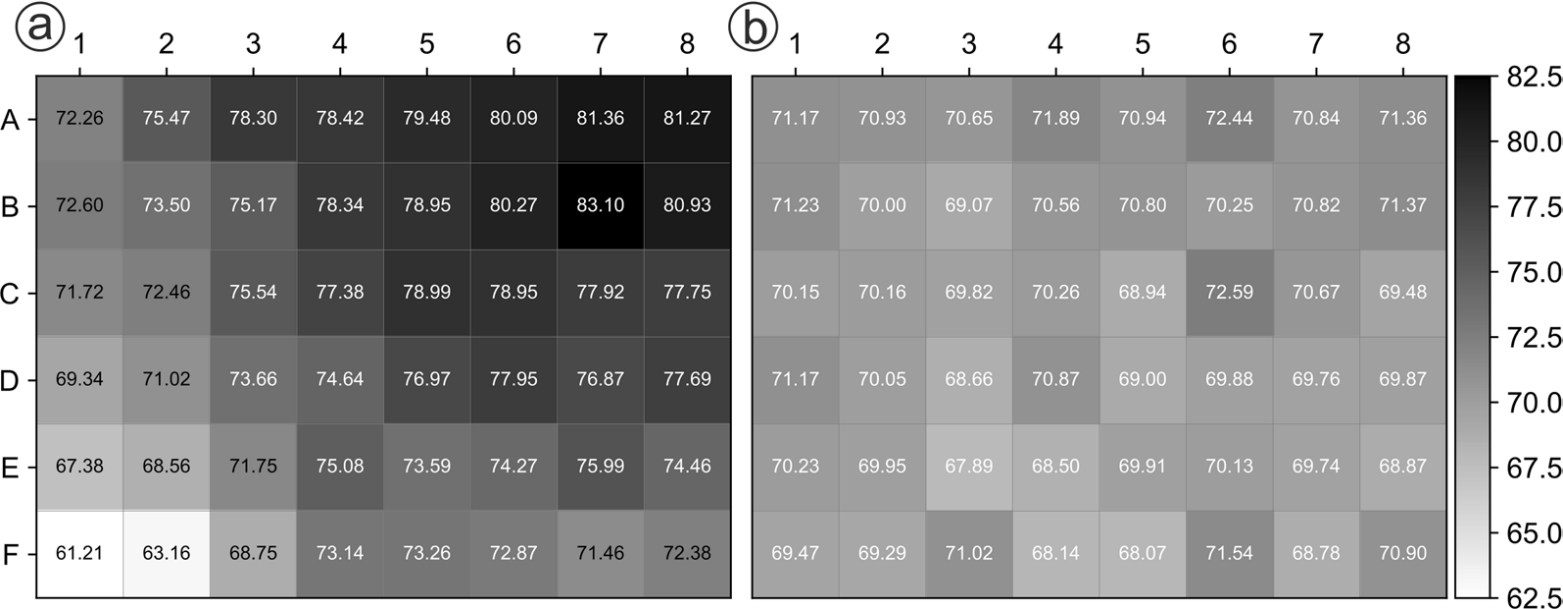
Position effect in a FlowerPlate with 48 replicates of *E. coli* BL21(DE3) + *Bs*GDH-PT-CBDCell during BioLector cultivation. The cultivations were performed for 72 h at **a:** 3 h 37 °C and 69 h 15 °C or **b:** at 25 °C in 1 mL M9-AI medium with 1000 rpm. The illustrated numbers in each well symbolize the backscatter values in a.u. at the end of the cultivation

As a starting point before optimization, a standard microcultivation protocol for CatIBs was taken from literature, which comprised two cultivation phases with 37 °C for 3 h and 15 °C for 69 h, respectively [30]. The first challenge that was noted was a systematic bias over the positions in the BioLector, which was observed during the second cultivation step at 15 °C (Fig. 3a, see “Automated protein production and protein purification” section). For further investigation of this effect, we cultivated 48 biological replicates of *E. coli* BL21(DE3) with *Bs*GDH-PT-CBDCell, a variant that was previously identified as a positive CatIB producer under standard conditions in a manual study [11]. Strikingly, the final backscatter values across the FlowerPlate varied from approximately 60 to 80 a.u. with a mean and standard deviation of 74.9 a.u. and 4.5 a.u. respectively. Whereas the lowest values were reached in the bottom left area of the FlowerPlate, the highest values were observed in the top right area. This strongly indicates that the temperature switch from 37 °C to 15 °C, as was previously published as optimal conditions [30], causes a temperature gradient in the FlowerPlate, resulting from a corresponding gradient in the cultivation chamber of the BioLector. This is substantiated by the fact that the cooling fan outlet is placed in the bottom left part of the cultivation chamber. The resulting broad range of backscatter values would have an influence on biomass growth and formation of CatIBs, making it difficult to find the best performing CatIB producer. Variants that grow in the bottom left area are likely to always perform worse than variants from the top right area. A screening under these cultivation conditions would lead to an unfair ranking of the CatIB variants and therefore had to be optimized.

In order to confirm the hypothesis that temperature shift is the main reason for the high variability in final backscatter values, the cultivation temperature was changed to 25 °C for the whole process, excluding the cooling step (Fig. 3b). In this experiment, a similar final backscatter of 70.2 ± 1.1 a.u. was observed for 48 biological replicates at the end of the cultivation. As indicated by the strongly reduced standard deviation, the positional bias could thus be prevented by using 25 °C as cultivation temperature.

After adapting the cultivation temperature to 25 °C, an analysis was performed to test whether CatIBs were still formed and also if they retain enzymatic activity. In a first step, all strains with different *Bs*GDH-CatIB variants were cultivated under CatIB standard conditions (3 h at 37 °C, 69 h at 15 °C) or at 25 °C for 72 h in the modified approach. After cultivation, microscopic images of each strain variant were taken (Additional file 1: Figure S8). Moreover, the number of CatIB producing cells were determined to visualize the influence of the cultivation conditions. Interestingly, the comparison of both cultivation temperatures showed that different CatIB variants have different preferred cultivation conditions [31]. Although the activity is dependent on the specific variant, the results indicate that cultivation at 25 °C overall has a positive influence on the CatIB formation rate for the *Bs*GDH case study. In this study, three variants (*Bs*GDH-PT-3HAMP, *Bs*GDH-PT-CBDCell, *Bs*GDH-PT-18AWT) were additionally tested for their specific volumetric activity P_v_ (Additional file 1: Figure S9), indicating favorable activities at 25 °C. The optimized cultivation conditions were thus used in the following screening experiments.

### Optimization of the CatIB workflow – Enzymatic assay and overall validation

Besides standardizing and optimizing the cultivation conditions, the enzymatic assay was adapted compared to a recent manual study [11]. Previously, samples were taken after 0, 3, 6, 12 and 20 min from the *Bs*GDH reaction and quenched with methanol to stop the reaction. Inactivated samples were then diluted, transferred to a microtiter plate and NADH fluorescence was determined in a photometric measurement. However, sampling and subsequent transfer to new microtiter plates that can be measured in the plate reader leads to low data density and complex optimization of liquid handling. In comparison, online measurement of the reaction in the plate reader could drastically increase the resolution of data. In addition, pre-warming of solutions was performed to avoid any temperature effects during the NADH measurement (see “Automated activity assay” section).

Accordingly, the next step was a validation study to assess the reproducibility of the complete workflow from microcultivation of *E. coli* CatIB producers to the optimized online enzymatic assay (Additional file 1: Figure S2). Here, a high reproducibility of the new workflow was demonstrated with 42 biological replicates, lowering the relative standard deviation of fluorescence signals after 120 min to only 1.9% compared to 11.4% in a previous study [31]. Overall, this was achieved after (I) changing the cultivation temperature to eliminate the positional bias in the BioLector, (II) adapting the liquid handling settings and introducing pre-warming of assay solutions and (III) changing to online measurements for a higher data resolution. Whereas the manual processing and testing of 48 CatIB variants would require approximately 40 h of work time, the implemented, optimized and validated workflow for the screening of 48 CatIB variants could be performed in less than 11 h, including approximately 1 h manual workload. Therefore, the robotic automation resulted in a time saving of manual work by more than 90%. Together with construction of CatIBs, this results in an overall manual work time of 7 h for 48 variants. This is approximately 12% of the operator workload for the typical manual procedure, representing a great advance in testing and application of CatIBs. Due to the successful automation of the CatIB screening, which enables high-throughput testing in the context of DBTL cycles, the workflow could be applied to analyze all 63 *Bs*GDH-CatIB variants.

### Automated screening of 63 *Bs*GDH-CatIBs via Thompson sampling

In the final part of this work, the established tools for automated cloning, cultivation and characterization of CatIBs were combined in a case study with 63 *Bs*GDH-CatIB variants and *Bs*GDH_WT_. Since cultivation in FlowerPlates is limited to a maximum of 48 strains in parallel (or even less in case of biological replicates), a suitable design strategy for iterative screening rounds was to be identified, addressing the well-known exploration–exploitation dilemma. Here, the goal was to identify the best candidates in as few experiments as possible (exploitation), while ensuring that the library was sufficiently screened for promising variants that might improve the best candidate seen so far (exploration). We used two tools to achieve this goal: (i) we established a suitable, probabilistic process model that can describe the CatIB kinetic reaction and (ii) we applied Thompson sampling as a decision strategy to design experimental layouts for replicates, balancing exploration and exploitation in iterative screening rounds.

The process model is described in more detail in “Process model” section. Essentially, the probabilistic model represents the reaction in the *Bs*GDH assay (Additonal file 1 - Figure S2) as a first order reaction, where NADH is increasing in an exponential reaction with a constant reaction rate ${k}_{{\text{assay}}}$. However, the apparent rate in the assay is influenced by several factors such as the dilution factor, pipetting errors and the time delay in the reaction start due to the consecutive pipetting of individual columns. Since a good ranking should be based on the reaction rate of the CatIB variant, in the following denoted as ${k}_{{\text{variant}}}$, instead of the apparent reaction rate in the assay, it is important that the process model takes different errors and time delays into account to quantify these factors correctly. The model is inspired by a similar approach that was already successfully applied in the screening of PET-degrading enzymes [24]. We applied a Bayesian model, meaning that it yields probability distributions for each model parameter, thus quantifying the uncertainty of estimates.

In order to rank strains by activity of produced CatIBs, which can be influenced both by the number of CatIBs and their specific activity, the reaction rate of each CatIB variant given the experimental data is a suitable aggregate KPI. For reasons of high throughput and automation, counting of CatIBs or manual determination of CatIB mass was omitted in the screening process, which would be required to screen based on a specific activity. Instead, the aggregate KPI can be obtained directly from the process model, which provides probability distributions of each ${k}_{{\text{variant}}}$. As a remaining task, a suitable strategy to choose replicates for each round of experiments is required, which can be phrased as an optimization problem. For this, we applied the strategy of Thompson sampling, a well-known strategy for Bayesian optimization [27, 32, 33].

**Fig. 4:**
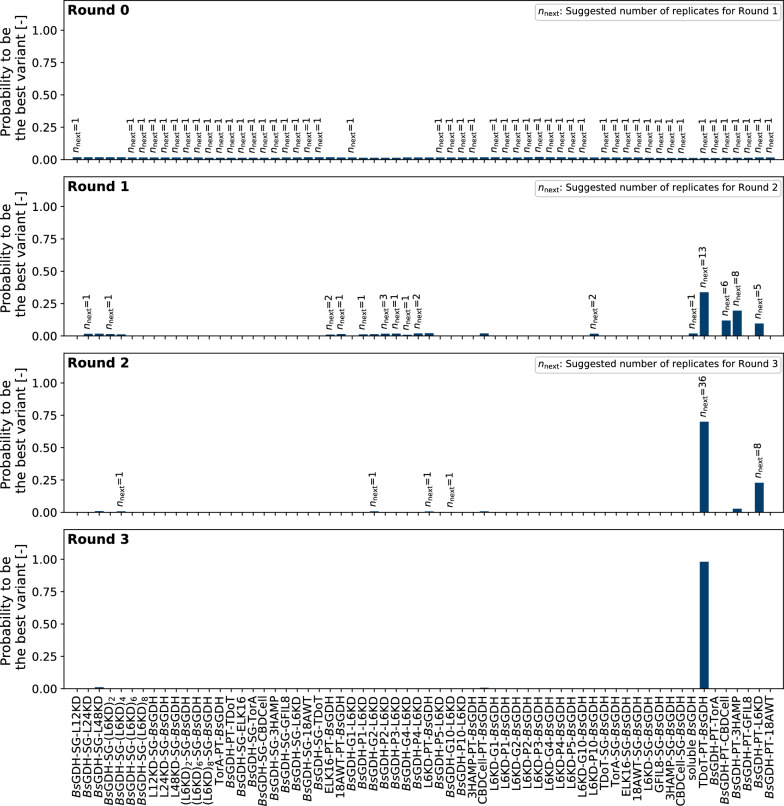
Probabilities of each of the 63 *Bs*GDH-CatIB variants (plus soluble *Bs*GDH as negative control) to be the best candidate. The probabilities are obtained using the sampling_probabilities function of the pyrff Python package, which essentially repeats Thompson sampling with several thousand samples from the probability distributions. The percentage in which a candidate was chosen by Thompson sampling is plotted as the probability to be the best variant in the library. Before any data is collected (Round 0, top), all variants are given the same prior probability for their reaction rate ${{\varvec{k}}}_{\mathrm{v}\mathrm{a}\mathrm{r}\mathrm{i}\mathrm{a}\mathrm{n}\mathrm{t}}$, which can be seen by the even distribution of probabilities. The number n above the bars indicates how often a variant was suggested by the algorithm for the upcoming round

In short, instead of using standard methods such as a triplicate design for each variant, Thompson sampling suggests designs based on the probability distributions of a KPI, in our case the reaction rate ${k}_{{\text{variant}}}$. In case of great uncertainty and thus wide distributions of the KPI, variants have a higher chance to be chosen since there is potential for improvement. On the other hand, variants with a narrow probability distribution and lower reaction rates ${k}_{{\text{variant}}}$ will be omitted in the next screening round. The algorithm therefore balances between exploring in uncertain areas and exploiting knowledge from previous rounds. Combined with the process model to evaluate data after each screening round, this algorithm was applied in three consecutive experiments, after each of which the new variant design was generated (Fig. 4).

The graph shows the probability of each variant to be the best candidate in the library after the indicated screening round, based on the current estimation of the KPI. Round 0 denotes the probabilities before any experimental data was measured, also called the prior probabilities. The probabilities on the y-axis are obtained by repeatedly applying the Thompson sampling algorithm on correlated samples from the probability distributions of each reaction rate. It was then counted how often a candidate was chosen given the overall number of draws (see “Thompson sampling” section). For Round 0, it can be observed that all variants had the same probability to be the best in the library, which reflects that no a priori knowledge exists for the given library. The algorithm accordingly drew candidates for the next FlowerPlate cultivation randomly, where the choice for the next round is indicated by the numbers above the bars. The choice of single replicates by the algorithm is a clear benefit in contrast to standard experimental designs, which commonly require multiple replicates.

The benefits of Thompson sampling can be seen from Round 1 on, where the probabilities are updated based on the measured data of the unicates. First, it can be observed that several variants such as the different (L6KD)_n_-SG-*Bs*GDH constructs dropped to a probability of 0% already after one round of experiments. This is the consequence of no activity in the respective NADH assay, indicating that no CatIBs were formed for the variant (compare to Fig. 6). In the scheme of exploitation, the algorithm decided not to assign further replicates to these variants as it would be done in a triplicate design. Instead, a mixture of unseen variants (e.g., *Bs*GDH-SG-L24KD) and the most promising so far (TDoT-PT-*Bs*GDH, *Bs*GDH-PT-CBDCell, *Bs*GDH-PT-3HAMP, *Bs*GDH-PT-L6KD) was chosen for Round 2, which is indicated by the number above the bars of Round 1. After Round 2, a greater shift towards exploitation can be seen as 36 replicates are assigned to the most promising variant TDoT-PT-*Bs*GDH for Round 3, already indicating converging behavior.

Moreover, the probability for unseen variants such as *Bs*GDH-SG-L48KD dropped to around 1% in Round 2. This is caused by the hierarchical structure of the process model (see “Process model” section). Since the expectation of the mean and standard deviation of the whole library was learned from the screening data and TDoT-PT-*Bs*GDH clearly lay above this expectation, it became less and less likely that unseen variants could still outperform this CatIB candidate. Accordingly, out of the six unseen variants *Bs*GDH-SG-L48KD, *Bs*GDH-SG-(L6KD)_4_, *Bs*GDH-G2-L6KD, *Bs*GDH-G10-L6KD, L6KD-PT-*Bs*GDH and CBDCell-PT-*Bs*GDH, only four were chosen for the third and final screening round. Afterwards, TDoT-PT-*Bs*GDH showed a 98% probability to be the best performer in the library (see Round 3), which exceed the termination criterion of > 95% probability. Overall, the top producer TDoT-PT-*Bs*GDH was measured in 50 replicates, while the second- and third-best variants were assigned nine and seven replicates, respectively. This led to a statistically sound characterization of the top producers in three rounds only, saving approximately 25% of experimental capacity and resources. Given the 64 variants (63 in the library and *Bs*GDH_WT_), a triplicate design would have led to 64 variants * 3 replicate per variant / 48 replicates per round = 4 rounds, i.e., an additional experimental round would be required. However, due to biological variance, pipetting errors and measurement noise, a higher number of replicates is needed to determine the top producer with 95% probability. Screening all variants with such a high number of replicates is unfeasible, thus demonstrating that Thompson sampling has great advantages by combining a statistically robust screening with exploitation of knowledge after each round, thus reducing replicates for bad CatIB producers and assigning them to the good producers instead.

**Fig. 5:**
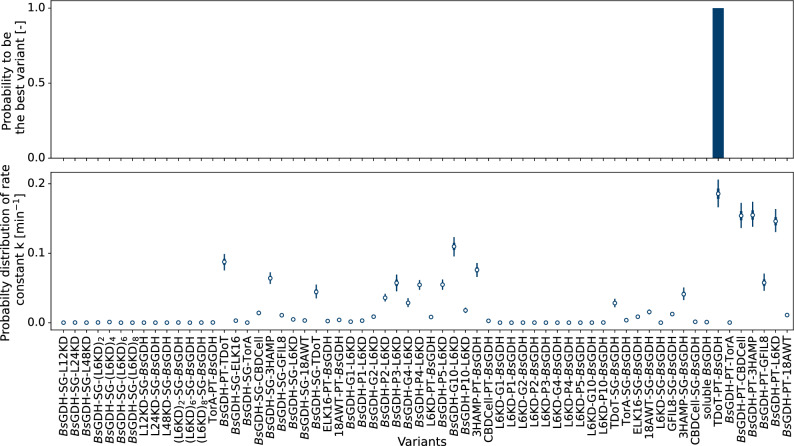
Probability of each variant to be the best candidate in the library after a manually designed fourth screening round (top). The top ten candidates as well as two previously unseen variants, *Bs*GDH-SG-L48KD, and CBDCell-PT-*Bs*GDH, were screened in four replicates each. The final ranking (bottom) shows that the top four candidates have similar reaction rate constants ${k}_{{\text{variant}}}.$ The white dot indicates the median of the distribution while thicker tails show the range in which 50% of the probability distribution lies. The thinner part of the tails spans the range to 94% probability

Due to the probabilistic nature of the algorithm, two remaining variants, *Bs*GDH-SG-L48KD, and CBDCell-PT-*Bs*GDH, where not chosen in any round. While the decision is statistically sound, it might be unsatisfying for a human operator. To confirm the results of the screening and challenge the decision policy of the algorithm, a fourth round was thus manually designed, in which the two unseen variants as well as the top ten candidates were measured in four replicates each (Fig. 5).

After the fourth round, it is clearly confirmed that TDoT-PT-*Bs*GDH is the best performer in the library, which the algorithm determined with a probability of > 99% (Fig. 5, top). As a matter of fact, the previously unseen variants *Bs*GDH-SG-L48KD and CBDCell-PT-*Bs*GDH did not lead to the successful formation of CatIBs and were weak performers not worth testing, thus confirming the low expectation of the algorithm given the mean rate constant of the whole library. While the probability to be the best variant is illustrative to explain how Thompson sampling is choosing replicates, it does not reveal how close variants are in their determined reaction rate constant ${k}_{{\text{variant}}}.$ The ranking given the KPI for all 64 variants is thus shown in (Fig. 5, bottom).

Here, the top four candidates, TDoT-PT-*Bs*GDH, *Bs*GDH-PT-3HAMP, *Bs*GDH-PT-CBDCell and *Bs*GDH-PT-L6KD all have a median reaction rate constant between 0.15 min^−1^ and 0.19 min^−1^ (white dots). Such close rates make it harder to distinguish the top producer since it does only exceed the activity of the other candidates by a small margin. However, the high number of replicates leads to narrow probability distributions in the estimation, which is indicated by the short tails in the forest plot (Fig. 5, bottom), showing the range in which the rate constants lie with 94% probability, similar to a confidence interval for frequentist statistics.

Overall, these experiments successfully demonstrate how a combination of automated workflow and model-assisted decision making can enhance screening in a powerful way. This is realized by not only reducing the number of experiments but at the same time achieving greater certainty in decisions. These insights will hold true especially for libraries of larger number of variants, where the benefit is expected to increase substantially. Please note that suitability of Thompson sampling is not limited to CatIB applications but could be used in selection processes of microbial production strains, proteins engineering or other targets, where resource efficiency and throughput is an issue. As a final step of this case study, the Thompson sampling results were compared to microscopy and SDS-PAGE data to shed light on the structure/function learnings.

### Insights on structure/function relationship

**Fig. 6:**
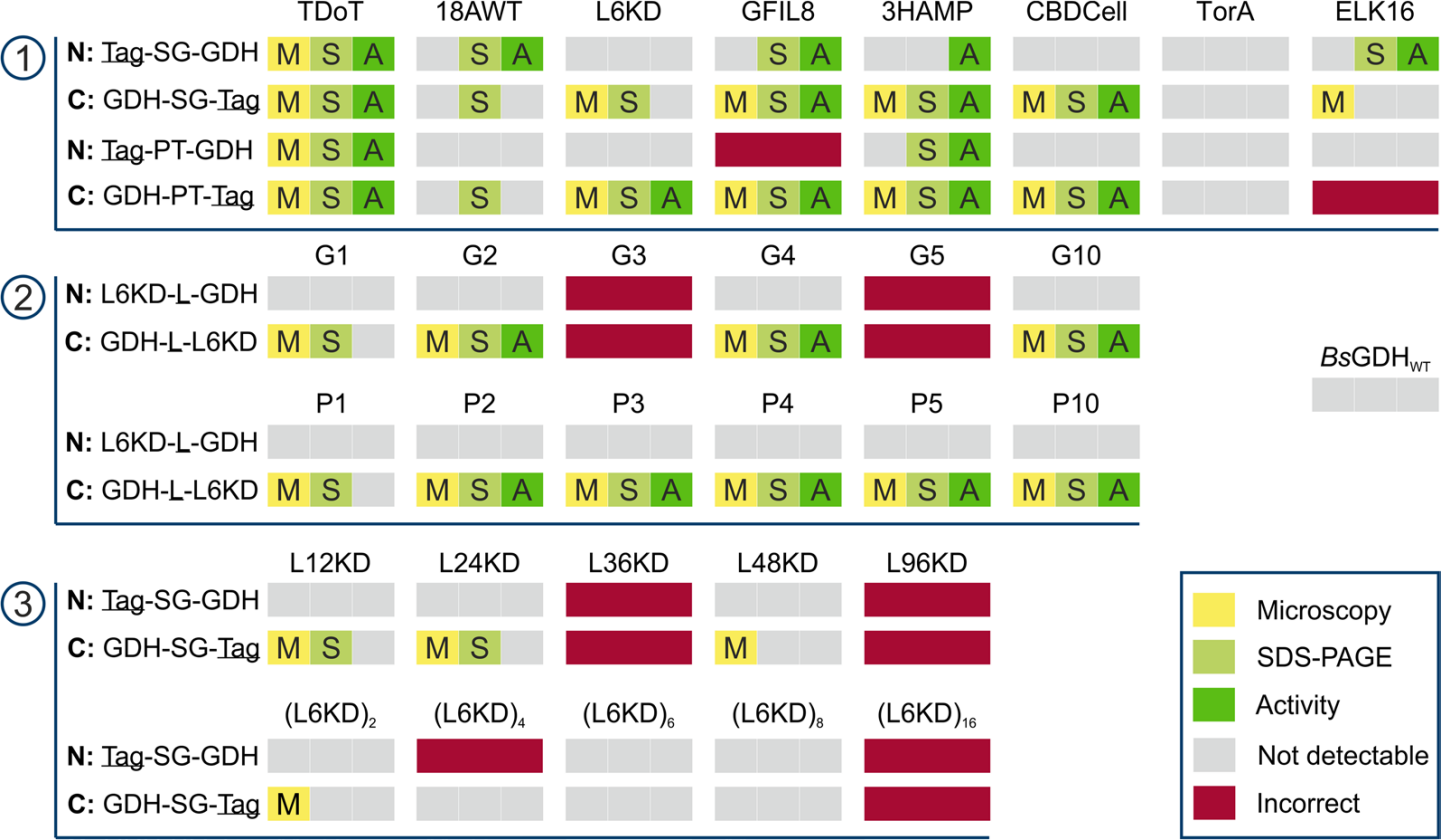
Overview of 63 *Bs*GDH-CatIB analyses using microscopy (yellow), SDS-PAGE (light green) and enzyme assay (dark green). The gray areas indicate no detection of CatIBs or enzymatic activity during microscopy, SDS-PAGE or enzyme assay. The red-marked constructs represent CatIB combinations that could not be correctly constructed and were thus not tested. **1** Testing of different linker/aggregation-inducing tag combinations **2** Linker study with different length of proline and glycine linkers **3** Tag study with different length of L6KD tag

In addition to the automated CatIB screening workflow, the formation of *Bs*GDH-CatIBs in the respective producer strain was visualized using phase contrast microscopy (Additional file 1: Figures S3-S6). Moreover, an SDS-PAGE analysis was performed to investigate the insoluble CatIB fraction after automated cell lysis and CatIB purification process (Additional file 1: Figure S7). The results are summarized in a qualitative manner in Fig. 6.

Several trends can be derived from Fig. 6. First, the control *Bs*GDH_WT_, which is expressing soluble enzyme, did not form visible inclusion bodies on microscopic images or during SDS-PAGE analysis. Moreover, the enzyme assay revealed that no activity was measured for the insoluble cell pellet fraction. These results indicate that *Bs*GDH_WT_ did not form natural CatIBs, which confirms previous results from a manual *Bs*GDH study [11].

Regarding the tag study (Fig. 6, panel 3), low to no CatIB formation was seen in microscopy (Additional file 1: Figure S6), showing that larger versions of the L6KD linker did not lead to better CatIB formation. In comparison, the linker study (Fig. 6, panel 2) included three of the best ten CatIB formation candidates of Thompson sampling. In total, 10 of the 20 CatIB candidates in the linker study formed visible CatIBs (Additional file 1: Figure S5). However, successful CatIB formation and activity highly depend on the combination of aggregation-inducing tag, length and type of the linker and N-/C-terminal fusion, allowing for no general conclusion, which is in line with the findings in [31].

The top four variants found in Thompson sampling, TDoT-PT-*Bs*GDH, *Bs*GDH-PT-3HAMP, *Bs*GDH-PT-CBDCell and *Bs*GDH-PT-L6KD, were all detectable via SDS-PAGE and microscopy (Fig. 6, panel 1). Consequently, it seems that the more rigid structure of the PT linker led to the most active CatIB variants in this case study. Regarding the position of the aggregation-inducing tag, C-terminal fusion was overall more beneficial, although successful CatIB variants were found for both fusion sides. This can also be seen for the linker study with proline and glycine linkers (Fig. 6, panel 2). These findings indicate beneficial structures with C-terminal fusion and PT linker that led to high CatIB production for *Bs*GDH, which is the goal of a DBTL cycle. Interestingly, however, the top performer TDoT-PT-*Bs*GDH is a variant with N-terminal addition of the tag, as such a perfect example of the still limited understanding of the structure–function relationship for CatIB design. These findings demonstrate again that enzyme-specific screening of linker and tag combinations seems to be required to identify the most efficient CatIB producers for each case. For this purpose, we demonstrated the usefulness of our toolbox, particularly the choice of Thompson sampling in screening of larger libraries.

## Conclusions

The aim of this study was to develop a CatIB toolbox towards a full DBTL cycle, which included automated strain construction, cultivation, microscopy, as well as CatIB purification and characterization, thus enabling the analysis of various CatIB candidates in parallel. The toolbox will contribute to finding the best performing CatIB variant of each enzyme and to get new insights of the CatIB structure–function relationship.

*Bs*GDH-PT-CBDCell was used to implement, optimize and validate the automated CatIB processing workflow. After (I) increasing the cultivation temperature to eliminate the position effect in the BioLector, (II) adaptation of the liquid handling settings and pre-warming of assay solutions and (III) online measurements for a higher data resolution, the validation study revealed a highly reproducible automated purification and enzymatic assay workflow with only 1.9% relative standard deviation across 42 replicates. For the whole CatIB toolbox, starting from strain construction until enzymatic analysis, only 7 h of manual work instead of 59 h were needed, which equals a reduction of 88% for 48 CatIB variants.

The automated workflow was used to analyze all 63 constructed *Bs*GDH-CatIBs in combination with Thompson sampling as a decision tool for strain selection for three rounds of FlowerPlate cultivations. Compared to a triplicate-based experimental design, which would have required four rounds to accommodate all replicates, the best candidate TDoT-PT-*Bs*GDH was identified in only three rounds with superior statistical certainty. Due to the exploitation-exploration balancing, the Thompson sampling algorithm selected this variant in 50 biological replicates during the three screening rounds, leading to a statistically sound estimation of its reaction rate constant as the KPI for a library ranking.

The analysis of *Bs*GDH-CatIBs revealed new insights in the CatIB structure–function relationship, in particular that the more rigid PT linker was again more likely to form successful CatIB variants compared to the SG linker. However, a priori prediction of necessary gene sequences for successful CatIB formation is still not possible. New target enzymes require different linker/aggregation-inducing tag combinations, which can be added N- or C-terminal. In conclusion, the presented CatIB toolbox is an important step to enhance and simplify the screening of large CatIB libraries, facilitating the identification of best CatIB producers.

In the future, development could be directed towards a feedback loop between process model and strain construction, indicating novel variants that should be constructed in the lab. Moreover, investigating both CatIB variants and cultivation conditions simultaneously could reveal further insights into successful and more reliable formation of CatIBs. For both goals, further automation of experimental procedures as well as data analysis pipelines will play an important role. This will ultimately allow the generation of sufficient data for extended structure–function studies, shedding light on the underlying relationship between the different building blocks for highly performant CatIBs in biocatalytic applications.

## Methods

### Reagents and chemicals

All chemicals were purchased from ROTH (Karlsruhe, Germany) and Merck (Sigma-Aldrich, Burlington, MA, US), unless stated otherwise. Enzymes for molecular biology were purchased from New England Biolabs GmbH (Frankfurt am Main, Germany).

### Preparation of competent cells

10 mL LB medium were inoculated with the respective strain and cultivated in 100 mL shake flasks at 37 °C and 170 rpm overnight. 1 mL of the preculture was transferred into 100 mL LB medium in 500 mL shake flasks. The shake flasks were incubated at 37 °C and 170 rpm until an OD_578nm_ of 0.6–0.8 was reached. The culture was subsequently filled into two 50 mL centrifuge tubes and centrifuged at 3300 x*g* and 4 °C for 10 ﻿min﻿. The supernatant was discarded and the cells were carefully resuspended with 10 mL ice-cold CaCl_2_ (7.35 g L^−1^)/glycerol (50 g L^−1^) solution. A second centrifugation was conducted with the same parameters and the supernatant was discarded. The cell pellets were resuspended with 1 mL CaCl_2_/glycerol solution in each centrifuge tube. The obtained suspensions in both centrifuge tubes were combined and aliquoted in prechilled, 1.5 mL reaction tubes, containing 0.5 mL each. The reaction tubes were immediately frozen and stored at – 80 °C. All steps were conducted on ice and in a sterile environment to avoid contamination.

### Automated construction of expression plasmids

The synthetic gene of the *Bs*GDH, the SG, PT, G1, G2, G3, G4, G5, G10 or P1, P2, P3, P4, P5 and P10 linker, as well as the aggregation-inducing tags, TDoT, 18AWT, L6KD, GFIL8, 3HAMP, TorA, CBDCell, ELK16, L12KD, L24KD, L36KD, L48KD, L96KD, (L6KD)_2_, (L6KD)_4_, (L6KD)_6_, (L6KD)_8_, (L6KD)_16_, were synthesized by Synbio Technologies (Monmouth Junction, NJ, US). The synthetic sequences contained *Bsa*I recognition and restriction sites needed for GGA. The synthetic gene encoding for *Bs*GDH was assembled with one of the linkers, one aggregation-inducing tag as well as the so-called suicide plasmid in a ratio of 1:1:1:3 during GGA. The suicide plasmid served as the expression plasmid backbone. It consisted of a pET28a(+) vector carrying the *ccdB* gene, coding for the CcdB toxin, which is lethal for standard *E. coli* strains, such as *E. coli* DH5α and *E. coli* BL21(DE3). It functioned as an accurate GGA control with zero-background cloning [34]. During GGA, the *ccdB* gene was removed by *Bsa*I and the CatIB linker-tag sequence was inserted by the T4 ligase. After *E. coli* DH5α was transformed with the GGA mixture, only strains carrying the successful CatIB plasmid were able to grow while strains carrying the original vector were killed due to the produced toxin. To start GGA, 2.5% (v/v) T4 ligase (400,000 U mL^−1^) as well as 2.5% (v/v) *Bsa*I restriction enzyme (20,000 U mL^−1^), was added to the mixture (Additional file 1: Table S3). The GGA was performed in a PCR cycler (37 °C, 1 min and 16 °C, 5 min–15 cycles; 85 °C, 20 min). Information about all plasmids that were used in this study are summarized in Additional file 1: Table S1. The final expression plasmids were sequenced and verified for the correct assembly (Eurofins GmbH, Hamburg, Germany).

### Heat shock transformation

For manual transformation, 20 µL of plasmid DNA were transferred to a 1.5 mL reaction tube and 80 µL of competent *E. coli* cells were added. The reaction tube was stored on ice for 20 min and incubated at 42 °C in a ThermoMixer® (Eppendorf SE, Hamburg, Germany) for exactly 30 s. After heat shock, the samples were stored on ice for 2 min. To regenerate the transformed cells, 1 mL of super optimal medium with catabolic repressor (SOC) medium was added and the reaction tube was incubated at 37 °C and 300 rpm for 1 h. The cells were plated on LB agar plates with the appropriate antibiotic. LB agar plates were incubated overnight at 37 °C.

Automated retransformation was conducted with the OT-2 lab robot with the 20 µL and 300 µL pipettes (Opentrons, Long Island City, NY, US) using an integrated thermocycler module. Unlike manual transformation, the automated transformation was conducted in a 96-well microtiter plate with lower volumes. 5 µL of plasmid DNA were added to 25 µL of competent *E. coli* cells. The first cooling step (20 min at 4 °C), the heat shock (30 s at 42 °C) and the second cooling step (2 min at 4 °C) were conducted in the thermocycler module. 170 µL LB medium were transferred to each well and the 96-well microtiter plate was incubated at 37 °C for 30 min in the thermocycler module. Afterwards, 5 µL of the suspension were plated on LB agar medium with the appropriate antibiotic utilizing the automated pipetting setup of the platform. LB agar plates were incubated overnight at 37 °C.

### Plasmid preparation

All plasmid preparations were carried out with the NucleoSpin® Plasmid (NoLid) kit (Macherey–Nagel, Düren, Germany). To obtain a sufficient amount of plasmid, the respective *E. coli* strains were cultivated in 2.5 mL LB medium with the appropriate antibiotic in a 5 mL square-well plate (Ritter GmbH, Schwabmünchen, Germany). The plasmid preparation was conducted with 2 mL culture broth according to the manufacturer description for the isolation of high-copy plasmid DNA from *E. coli* of the NucleoSpin® Plasmid kit (Macherey–Nagel, Düren, Germany). All steps in which the supernatant was later discarded were conducted utilizing a vacuum pump (Büchi, Essen, Germany) with a NucleoVac 24 Vacuum Manifold (Macherey–Nagel, Düren, Germany) for the NucleoSpin® Plasmid kit columns.

### Automated protein production and protein purification

CatIB production was performed by cultivating *E. coli* BL21(DE3) carrying the respective expression plasmids in M9 autoinduction medium (see Additional file 1: Table S2). The 1 mL main cultivation was performed at 25 °C for 72 h in a FlowerPlate in a BioLector I and was inoculated with an OD_600nm_ of 0.1 of an overnight culture in LB complex medium with additional kanamycin (37 °C, 1000 rpm). For comparison to the CatIB standard procedure, cultivation took place at 37 °C for 3 h, followed by 69 h at 15 °C. The purification process was continued with 650 µL cell suspension. After harvesting the cells from the BioLector, the cells were washed with 650 µL 0.9% (w v^−1^) sodium chloride solution. The cell lysis was performed with 500 µL cell lysis buffer (BugBuster® HT Protein Extraction Reagent (Merck KgaA, Darmstadt, Germany) with the addition of 0.146 g L^−1^ lysozyme) and the CatIB washing step afterwards was performed with 760 µL Milli-Q® water. During cell lysis, the samples were incubated on a BioShake® for 20 min at 1000 rpm and 20 °C. All centrifugation steps were performed at 3730 x*g* and 4 °C. The first two centrifugation steps for cell separation from cultivation broth and cell washing with NaCl were carried out for 15 min. In contrast, the last two centrifugation steps after cell lysis and CatIB washing with Milli-Q® water were performed for 30 min.

### Automated activity assay

To determine the enzymatic activity of *Bs*GDH-CatIB variants, the fluorescence of the formed NADH was measured with a photometer (Tecan, Männedorf, Switzerland). An excitation wavelength of 340 nm and an emission wavelength of 470 nm were chosen to measure NADH. The enzyme assay solution contained 40 mM TAE buffer (pH 7), 200 mM glucose and 0.4 mM NAD^+^. The assay solution and the purified CatIB suspension were pre-heated to 40 °C. After combining these two solutions, 250 µL of the mixture were incubated at 37 °C in a UV microtiter plate in a photometer to enable online measurement of the formed NADH during the reaction. For pipetting, plate transfer and pre-heating steps, the liquid handling and robotic manipulator arms, as well as the shaking/heating devices of the robotic platform Freedom Evo200 (Tecan, Männedorf, Switzerland), were used. A calibration curve with different NADH concentrations ranging from 0 mM to 0.25 mM was used to calculate the NADH concentration. This exponential relationship was fitted using the calibr8 Python package [35]. The fitted calibration model is shown in Additional file 1: Figure S10.

### Sodium dodecyl sulfate–polyacrylamide gel electrophoresis (SDS-PAGE)

For SDS-PAGE analysis, sample preparation was performed by adding 2 × Laemmli sample buffer to a purified CatIB-water suspension. After incubation for 10 min at 95 °C, the samples were applied to Criterion™ 4–12% Bis–Tris protein gel, 1.0 mm, with 18 wells (Bio-Rad Laboratories GmbH, Feldkirchen, Germany), together with a protein marker (PageRuler Prestained Protein *ladder*, ThermoFisher Scientific Inc., Waltham, MA, US). Gel electrophoresis was performed in NuPAGE™ MES SDS running buffer (1 ×) at 200 V, 500 mA and 150 W. The gel was stained with Simply Blue™ SafeStain for 1 h.

### Microscopic analysis

Phase-contrast microscopic analysis was performed for *E. coli* BL21(DE3) strains with CatIB formation. 1 µL of each sample was transferred to a microscope slide and covered with a coverslip. The microscope slide was positioned upside down on the desk of an inverted Nikon Eclipse Ti2 microscope (Nikon GmbH, Düsseldorf, Germany). The sample was observed with a CFI Plan Apo Lambda 100X Oil objective (Nikon GmbH, Düsseldorf, Germany) and images were taken with a Thorlab camera DCC154M-GL (Thorlabs Inc., Newton, NJ, US). To determine the percentage of CatIB-producing cells during the cultivation process, cells with visible inclusion bodies at the poles were manually counted (n_cells_ = 45–200).

### Software

All analyses and plots presented in this study were performed with recent versions of Python 3.8, PyMC == 4.0.0b2 [36, 37], ArviZ ≥ 0.11.4 [38], matplotlib ≥ 3.5 [39], NumPy ≥ 1.21 [40], pandas ≥ 1.4 [41, 42], SciPy ≥ 1.7 [43] and related packages. For calibration models, the in-house developed, publicly available calibr8 package was used with versions ≥ 6.5 [35, 44]. For parsing of BioLector data, the bletl package [45, 46] was applied. Photometric measurements were analyzed using the in-house developed retl package (not published). The robotools Python package was used to facilitate multi-step liquid-handling instructions on the robotic platform [47]. For a full list of dependencies see the accompanying GitHub repository [48].

### Process model

**Fig. 7:**
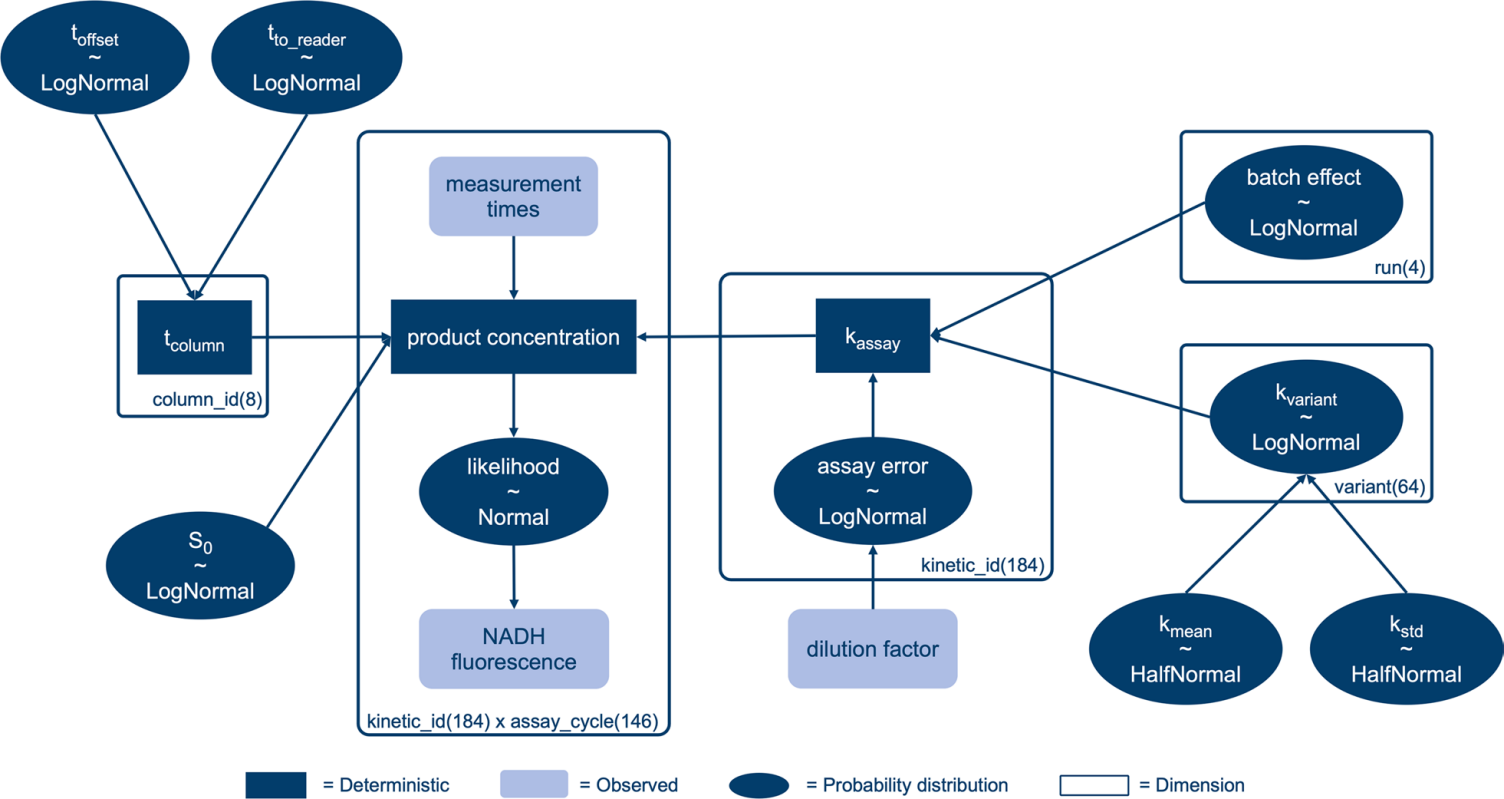
Graphical representation of the process model. The dark blue ovals indicate probability distributions for a parameter while squares are deterministic combinations of parameters. The empty boxes surrounding groups of parameters indicate the dimensionality of the resulting distributions, where column_id refers to the eight columns in the microtiter plate of the assay and kinetic_id to the unique combination of well and run. Light blue boxes indicate data that can be experimentally observed. The KPI used for ranking is the rate constant of each variant, ${k}_{{\text{variant}}}$ (center right). The two parameters below, ${k}_{{\text{mean}}}$ and ${k}_{{\text{std}}}$ , are hyperpriors that describe the expectation for mean and variance of kinetic rate constants for the whole CatIB library

The process model for this study closely follows the terminology and methods published in a recent study [24]. Briefly, a probabilistic model was created using PyMC v4, more precisely a Bayesian hierarchical model. Bayesian statistics allows to combine data-driven likelihoods with prior knowledge of the underlying process and its parameters. The resulting updated probability distributions of each parameter are called posterior probabilities. In this study, the process model was used to quantify various experimental effects and their influence on the reaction rate of each CatIB variant in the final activity assay (see “Automated activity assay” section). A computation graph of the model is shown in Fig. 7.

Round shapes in the computation graph represent probability distributions for a parameter, where the prior assumption is indicated by the sign ~ . Boxes with rounded edges represent observables while the rectangles show deterministic variables, i.e., distributions that can be calculated from the other variables. Finally, the surrounding boxes indicate the dimensionality of the different variables, e.g., being specific for a run, variant or a reaction well (kinetic_id). First, the product concentration (center left) should be focused on. For a ranking of the different CatIB variants, we chose to model their reaction in the activity assay with a first order mass action law, which results in the following equation for product formation:

#### ${P}_{t}={S}_{0} \bullet \left({1-e}^{{k}_{{\text{assay}}} \bullet {t}_{{\text{column}}}}\right)$,

with ${P}_{t}$ as the product concentration at time ${t}_{{\text{column}}}$, ${S}_{0}$ as initial substrate concentration and ${k}_{{\text{assay}}}$ as the rate constant in a well during a specific run. Due to column-wise pipetting, which is modeled as ${t}_{{\text{offset}}}$, as well as additional time between reaction start and positioning of the microtiter plate in the photometric device (${t}_{{\text{to}}\_{\text{reader}}}$), the time of reaction is specified per column as ${t}_{{\text{column}}}$. As shown in the computation graph, the rate constant ${k}_{{\text{assay}}}$ is influenced by the reaction rate of the CatIB variant, ${k}_{{\text{variant}}}$, which we assume to be specific for the variant under reproducible reaction conditions. As such, this variable serves as the KPI. However, batch effects between runs and the error of the assay itself, which is dependent on the dilution factor of the sample, influence the reaction rate that is finally observed. These different effects are combined into the final reaction rate ${k}_{{\text{assay}}}$, which is the variable that can be experimentally determined. As a consequence, the Bayesian model learns about the probability distributions of experimental errors over time while using a KPI for ranking that is only dependent on the biological differences, i.e., the variants themselves (Fig. 7).

To additionally learn about the expectation of the mean and standard deviation of the whole library, so-called hyperpriors ${k}_{{\text{mean}}}$ and ${k}_{{\text{std}}}$ were introduced. Essentially, the distributions for the mean and standard deviation of the population were derived from experimental data over the rounds. The prior distribution for each variant, i.e., the prior belief in the reaction rate ${k}_{{\text{variant}}}$, was chosen as a Normal distribution that is parameterized by ${k}_{{\text{mean}}}$ and ${k}_{{\text{std}}}.$ In practice, lower values for the mean of the whole population (e.g., because many CatIBs with very low activity were measured) also determine the expectation for an unseen variant, meaning that its prior distribution will have a lower mean value. For the choice of candidates for screening, such an unseen candidate would have a lower chance compared to already observed candidates with over-average reaction rates. Vice versa, should the population mean increase because many well-performing variants were observed, the prior of an unseen variant would increase as well, raising its chances to be selected by Thompson sampling for better exploration (see “Thompson sampling” section).

A mathematical notation of the model can be found in the Additional file 1. Additionally, the code for the process model can be found in the accompanying GitHub repository [48]. Posterior probabilities were obtained by Markov chain Monte Carlo sampling, using the No-U-Turn sampler [49] in PyMC. Convergence checks and inspection of the traces were performed using ArviZ.

### Thompson sampling

In our automated setup, 48 reaction wells could be used per batch experiments. To suggest the CatIB variants measured in each experiment, we used Bayesian optimization. The Bayesian process model yielded probability distributions for the estimated reaction rate of each variant, ${k}_{{\text{variant}}}$. Instead of using a fixed number of replicates for each variant, we used these distributions and Thompson sampling [50] for experimental design. More precisely, after each batch experiment, we updated the posterior probabilities of the process model with the new data. To draw a batch of 48 suggestions, we used the sample_batch function from the pyrff package [51] and applied it to the posterior probabilities of the rate constants. The suggested 48 candidates (allowing for replicates) were randomly assigned to cultivation wells for the next experiment.

By sampling from the distributions, Thompson sampling balanced exploration and exploitation naturally, i.e., allowing exploration for wide distributions with large uncertainty and excluding variants with low estimated reaction rates as means of exploitation. For a detailed tutorial on Thompson sampling, we refer to [27]. The experiment-model loop was interrupted when the model showed a probability of > 95% to have identified the top candidate in the library.

To obtain the probability of each candidate to be the best after the given round (Figs. 4, 5) we applied the sampling_probabilities function of pyrff, which essentially repeated Thompson sampling for correlated samples from the probability distributions and counted how often a candidate was chosen in the overall number of draws.

### Supplementary Information

**Additional file 1**: **Table S1**: List of all constructed plasmids. **Table S2**: Recipe of M9 Autoinduction medium – 1000 mL. **Table S3**: Recipe of Golden Gate Assembly Mix – 20 μL. **Figure S1**: Overview of 76 tested BsGDH-CatIB combinations and BsGDHWT control using semiautomated cloning workflow. **Figure S2**: NADH fluorescence of BsGDH-PT-CBDCell replicates as a validation study. **Figure S3**: Microscopic images of strains producing BsGDH-CatIBs with different linker/aggregation inducing tagcombinations tagged at the C-Terminus of the enzyme and BsGDHWT. **Figure S4**: Microscopic images of strains producing BsGDH-CatIBs with different linker/aggregation inducing tag combinations tagged at the N-Terminus of the enzyme. **Figure S5**: Microscopic images of strains producing BsGDH-CatIBs with different lengths of glycine or proline linker tagged at the C- and N-Terminus of the enzyme. **Figure S6**: Microscopic images of strains producing BsGDH-CatIBswith different lengths of L6KD tag linker tagged at the C- and N-Terminus of the enzyme. **Figure S7**: Evaluation of 63 BsGDH-CatIB formation and BsGDHWT by SDS-PAGE analysis. **Figure S8**: Influence of cultivation temperature on CatIB formation analyzed via microscopy. **Figure S9**: Influence of cultivation temperature on specific volumetric productivity of BsGDH-CatIBs. **Figure S10**: Calibration model that describes a normally distributed measurement error for measured NADH fluorescence in the assay.

## Data Availability

Screening data and Thompson sampling results are available in the accompanying Github repository via https://github.com/JuBiotech/Supplement-to-Helleckes-Kuesters-et-al.-2023. Further datasets used and/or analyzed during the current study are available from the corresponding author on reasonable request.

## Abbreviations

*Bs*GDH: Glucose dehydrogenase from *Bacillus subtilis*
CatIB: Catalytically active inclusion body
CatIB: Catalytically active inclusion body
DBTL: Design-Build-Test-Learn
DoE: Design of Experiments
GDH: Glucose dehydrogenase
GGA: Golden Gate Assembly
KPI: Key performance indicator
PCR: Polymerase chain reaction
PT: Proline/Threonine
SDS-PAGE: Sodium dodecyl sulfate polyacrylamide gel electrophoresis
SG: Serine/Glycine
SOC: Super optimal medium with catabolic repressor (SOC)
