# Supplementary material for: High-throughput screening of catalytically active inclusion bodies using laboratory automation and Bayesian optimization
Source: Microb Cell Fact. 2024 Feb 24;23:67. doi: 10.1186/s12934-024-02319-y (PMC10894497; doi:10.1186/s12934-024-02319-y)
Supplement: Supplementary file 1 — Additional file 1: Table S1: List of all constructed plasmids. Table S2: Recipe of M9 Autoinduction medium – 1000 mL. Table S3: Recipe of Golden Gate Assembly Mix – 20 μL. Figure S1: Overview of 76 tested BsGDH-CatIB combinations and BsGDHWT control using semiautomated cloning workflow. Figure S2: NADH fluorescence of BsGDH-PT-CBDCell replicates as a validation study. Figure S3: Microscopic images of strains producing BsGDH-CatIBs with different linker/aggregation inducing tagcombinations tagged at the C-Terminus of the enzyme and BsGDHWT. Figure S4: Microscopic images of strains producing BsGDH-CatIBs with different linker/aggregation inducing tag combinations tagged at the N-Terminus of the enzyme. Figure S5: Microscopic images of strains producing BsGDH-CatIBs with different lengths of glycine or proline linker tagged at the C- and N-Terminus of the enzyme. Figure S6: Microscopic images of strains producing BsGDH-CatIBswith different lengths of L6KD tag linker tagged at the C- and N-Terminus of the enzyme. Figure S7: Evaluation of 63 BsGDH-CatIB formation and BsGDHWT by SDS-PAGE analysis. Figure S8: Influence of cultivation temperature on CatIB formation analyzed via microscopy. Figure S9: Influence of cultivation temperature on specific volumetric productivity of BsGDH-CatIBs. Figure S10: Calibration model that describes a normally distributed measurement error for measured NADH fluorescence in the assay. [file 12934_2024_2319_MOESM1_ESM.pdf]

## Supporting Information

"High-throughput screening of catalytically active inclusion bodies using laboratory automation and Bayesian optimization"

Laura Marie Helleckes[1,2†], Kira Küsters[1,2†], Christian Wagner[1,2], Rebecca Hamel[1,2], Ronja Saborowski[1], Jan Marienhagen[1,2], Wolfgang Wiechert[1,3], Marco Oldiges[1,2*]

[†]These authors contributed equally.

[1]Institute of Bio- and Geosciences IBG-1: Biotechnology, Forschungszentrum Jülich GmbH, 52425 Jülich, Germany

[2]Institute of Biotechnology, RWTH Aachen University, 52074 Aachen, Germany

[3]Computational Systems Biotechnology (AVT.CSB), RWTH Aachen University, 52074 Aachen, Germany

*Corresponding author: m.oldiges@fz-juelich.de

ORCID-Identifier

Laura Marie Helleckes: https://orcid.org/0000-0001-7825-7998

Kira Küsters: https://orcid.org/0000-0002-2472-9926

Christian Wagner: https://orcid.org/ 0000-0002-8311-8412

Rebecca Hamel: https://orcid.org/0000-0002-2090-9364

Jan Marienhagen: https://orcid.org/0000-0001-5513-3730

Wolfgang Wiechert: https://orcid.org/0000-0001-8501-0694

Marco Oldiges: https://orcid.org/0000-0003-0704-5597

**Table S1:** List of all constructed plasmids. The sign "/" symbolize that each of the sequences were integrated into a vector backbone respectively. "N" and "C" symbolize the enzyme terminus that is fused with the linker and aggregation-inducing tag.

| Vector | Genotype |
|---|---|
| pET28a(+)::Kan | *ColE1 lacZ'* $Kan^R$ $P_{T7}$ $P_{lac}$ |
| pET28a(+)::Kan::CcdB | *ColE1 lacZ'* $Kan^R$ $P_{T7}$ $P_{lac}$ *ccdB* |
| pET28a(+)::Kan::*Bs*GDH_wildtype | *Bs*GDH_wildtype in pET28a(+) |
| pET28a(+)::Kan::*Bs*GDH_N/*Bs*GDH_C::SG::TDoT | *Bs*GDH_N/*Bs*GDH_C::SG::TDoT in pET28a(+) |
| pET28a(+)::Kan::*Bs*GDH_N/*Bs*GDH_C::SG::18AWT | *Bs*GDH_N/*Bs*GDH_C::SG::18AWT in pET28a(+) |
| pET28a(+)::Kan::*Bs*GDH_N/*Bs*GDH_C::SG::L6KD | *Bs*GDH_N/*Bs*GDH_C::SG::L6KD in pET28a(+) |
| pET28a(+)::Kan::*Bs*GDH_N/*Bs*GDH_C::SG::GFIL8 | *Bs*GDH_N/*Bs*GDH_C::SG::GFIL8 in pET28a(+) |
| pET28a(+)::Kan::*Bs*GDH_N/*Bs*GDH_C::SG::3HAMP | *Bs*GDH_N/*Bs*GDH_C::SG::3HAMP in pET28a(+) |
| pET28a(+)::Kan::*Bs*GDH_N/*Bs*GDH_C::SG::TorA | *Bs*GDH_N/*Bs*GDH_C::SG::TorA in pET28a(+) |
| pET28a(+)::Kan::*Bs*GDH_N/*Bs*GDH_C::SG::CBDCell | *Bs*GDH_N/*Bs*GDH_C::SG::CBDCell in pET28a(+) |
| pET28a(+)::Kan::*Bs*GDH_N/*Bs*GDH_C::SG::ELK16 | *Bs*GDH_N/*Bs*GDH_C::SG::ELK16 in pET28a(+) |
| pET28a(+)::Kan::*Bs*GDH_N/*Bs*GDH_C::PT::TDoT | *Bs*GDH_N/*Bs*GDH_C::PT::TDoT in pET28a(+) |
| pET28a(+)::Kan::*Bs*GDH_N/*Bs*GDH_C::PT::18AWT | *Bs*GDH_N/*Bs*GDH_C::PT::18AWT in pET28a(+) |
| pET28a(+)::Kan::*Bs*GDH_N/*Bs*GDH_C::PT::L6KD | *Bs*GDH_N/*Bs*GDH_C::PT::L6KD in pET28a(+) |
| pET28a(+)::Kan::*Bs*GDH_C::PT::GFIL8 | *Bs*GDH_C::PT::GFIL8 in pET28a(+) |
| pET28a(+)::Kan::*Bs*GDH_N/*Bs*GDH_C::PT::3HAMP | *Bs*GDH_N/*Bs*GDH_C::PT::3HAMP in pET28a(+) |
| pET28a(+)::Kan::*Bs*GDH_N/*Bs*GDH_C::PT::TorA | *Bs*GDH_N/*Bs*GDH_C::PT::TorA in pET28a(+) |
| pET28a(+)::Kan::*Bs*GDH_N/*Bs*GDH_C::PT::CBDCell | *Bs*GDH_N/*Bs*GDH_C::PT::CBDCell in pET28a(+) |

| | |
|---|---|
| pET28a(+)::Kan::*Bs*GDH_N::PT::ELK16 | *Bs*GDH_N::PT::ELK16<br>in pET28a(+) |
| pET28a(+)::Kan::*Bs*GDH_N/*Bs*GDH_C:: G1::L6KD | *Bs*GDH_N/*Bs*GDH_C::G1::L6KD<br>in pET28a(+) |
| pET28a(+)::Kan::*Bs*GDH_N/*Bs*GDH_C:: G2::L6KD | *Bs*GDH_N/*Bs*GDH_C::G2::L6KD<br>in pET28a(+) |
| pET28a(+)::Kan::*Bs*GDH_N/*Bs*GDH_C:: G4::L6KD | *Bs*GDH_N/*Bs*GDH_C::G4::L6KD<br>in pET28a(+) |
| pET28a(+)::Kan::*Bs*GDH_N/*Bs*GDH_C::G10::L6KD | *Bs*GDH_N/*Bs*GDH_C::G10::L6KD<br>in pET28a(+) |
| pET28a(+)::Kan::*Bs*GDH_N/*Bs*GDH_C::P1::L6KD | *Bs*GDH_N/*Bs*GDH_C::P1::L6KD<br>in pET28a(+) |
| pET28a(+)::Kan::*Bs*GDH_N/*Bs*GDH_C::P2::L6KD | *Bs*GDH_N/*Bs*GDH_C::P2::L6KD<br>in pET28a(+) |
| pET28a(+)::Kan::*Bs*GDH_N/*Bs*GDH_C::P3::L6KD | *Bs*GDH_N/*Bs*GDH_C::P3::L6KD<br>in pET28a(+) |
| pET28a(+)::Kan::*Bs*GDH_N/*Bs*GDH_C::P4::L6KD | *Bs*GDH_N/*Bs*GDH_C::P4::L6KD<br>in pET28a(+) |
| pET28a(+)::Kan::*Bs*GDH_N/*Bs*GDH_C::P5::L6KD | *Bs*GDH_N/*Bs*GDH_C::P5::L6KD<br>in pET28a(+) |
| pET28a(+)::Kan::*Bs*GDH_N/*Bs*GDH_C::P10::L6KD | *Bs*GDH_N/*Bs*GDH_C::P10::L6KD<br>in pET28a(+) |
| pET28a(+)::Kan::*Bs*GDH_N/*Bs*GDH_C::SG::L12KD | *Bs*GDH_N/*Bs*GDH_C::SG::L12KD<br>in pET28a(+) |
| pET28a(+)::Kan::*Bs*GDH_N/*Bs*GDH_C::SG::L24KD | *Bs*GDH_N/*Bs*GDH_C::SG::L24KD<br>in pET28a(+) |
| pET28a(+)::Kan::*Bs*GDH_N/*Bs*GDH_C::SG::L48KD | *Bs*GDH_N/*Bs*GDH_C::SG::L48KD<br>in pET28a(+) |
| pET28a(+)::Kan::*Bs*GDH_N/*Bs*GDH_C::SG::(L6KD)$_2$ | *Bs*GDH_N/*Bs*GDH_C::SG::(L6KD)$_2$<br>in pET28a(+) |
| pET28a(+)::Kan::*Bs*GDH_C::SG::(L6KD)$_4$ | *Bs*GDH_C::SG::(L6KD)$_4$<br>in pET28a(+) |
| pET28a(+)::Kan::*Bs*GDH_N/*Bs*GDH_C::SG::(L6KD)$_6$ | *Bs*GDH_N/*Bs*GDH_C::SG::(L6KD)$_6$<br>in pET28a(+) |
| pET28a(+)::Kan::*Bs*GDH_N/*Bs*GDH_C::SG::(L6KD)$_8$ | *Bs*GDH_N/*Bs*GDH_C::SG::(L6KD)$_8$<br>in pET28a(+) |

**Table S2**: Recipe of M9 Autoinduction medium – 1000 mL.

| | |
|---|---|
| Salt Stock solution (5x) | 200 mL |
| $MgSO_4*7H_2O$ solution (246.48 g $L^{-1}$) | 1 mL |
| $CaCl_2*5H_2O$ solution (14.702 g $L^{-1}$) | 1 mL |
| Trace element solution (1000x) | 1 mL |
| Citrate/Fe solution<br>(7.5 g L-1 $FeSO_4*7H_2O$<br>113.95 g L-1 tri-NaCitrat*$2H_2O$) | 2 mL |
| Thiamin solution (10 g $L^{-1}$) | 1 mL |
| 2 % (w/v) Lactose solution | 100 mL |
| 5 % (w/v) Glucose solution | 10 mL |
| Glycerin 99% | 4 mL |
| Kanamycin solution (50 g $L^{-1}$) | 1 mL |
| add Milli-Q water (final volume) | 1000 mL |
| | |
| **Salt Stock (5x)** | 1000 mL |
| $(NH_4)_2SO_4$ | 25 g |
| $KH_2PO_4$ | 15 g |
| $Na_2HPO_4$ | 33.9 g |
| NaCl | 2.5 g |
| $NH_4Cl$ | 10 g |
| add Milli-Q water (final volume) | 1000 mL |
| | |
| **Trace elements (1000x)** | 1000 mL |
| $AlCl_3*6H_2O$ | 0.75 g |
| $CoCl_2*6H_2O$ | 0.6 g |
| $CuSO_4*5H_2O$ | 2.5 g |
| $H_3BO_3$ | 0.5 g |
| $MnSO_4*1H_2O$ | 17.1 g |
| $Na_2MoO_4*2H_2O$ | 3 g |
| $NiCl_2*6H_2O$ | 1.7 g |
| $ZnSO_4*7H_2O$ | 15 g |
| Dissolve in 100 mL Milli-Q water and 50 mL 32% HCl, add Milli-Q water to final volume | |

**Table S3**: Recipe of Golden Gate Assembly Mix – 20 µL.

| **Component** | **Volume [µL]** |
|---|---|
| Backbone: pET28a(+)::Kan::CcdB | 6.0 |
| Vector insert 1: target enzyme | 1.0 |
| Vector insert 2: linker | 2.0 |
| Vector insert 3: tag | 1.0 |
| BsaI-HF®v2 | 0.5 |
| T4-ligase | 0.5 |
| T4-ligase reaction buffer (10x) | 2.0 |
| Milli-Q water | 7.0 |
| **Total volume** | **20.0** |

***Overview of semi-automated CatIB construction workflow***

To start the CatIB cloning process, plasmids containing the GGA fragments, i.e., the gene of interest, linker and aggregation-inducing tag sequence, were commercially synthesized by Synbio Technologies (Monmouth Junction, US). Competent *E. coli* DH5α and *E. coli* BL21(DE3) cells were prepared manually, because it was conducted only once in a large batch. The first transformation step of *E. coli* DH5α to multiply the copy number of the synthesized plasmid was automated using the Opentrons OT-2 liquid handling system together with an integrated thermocycler for heat-shocking and cooling of the cells. After transformation, 5 µL of the cells were spotted on an agar plate with the robotic platform. The transformed cells were used to purify the multiplied plasmid *via* an accelerated plasmid preparation by attaching the preparation filters on a vacuum station instead of performing several centrifugation steps. Automation of this step was challenging because plasmid amounts after automated purification were too low for efficient GGA. However, the acceleration of this step already led to a time-saving of approximately 20 %. The master mix for GGA was set up by mixing the required enzymes and buffer with Milli-Q® water. This short step was performed manually to ensure a remaining activity of the heat-sensitive reagents. GGA was performed again with the Opentrons OT-2 system in combination with the integrated thermocycler for the incubation of BsaI restriction enzyme or T4-ligase. The following transformation of *E. coli* DH5α with assembled GGA constructs was performed manually because the volumes for transformation in the integrated thermocycler was limited to 200 µL. However, to ensure successful transformation after GGA, a minimum of 1 mL batches were required. The following plasmid preparation and the retransformation of *E. coli* BL21(DE3) to generate the CatIB production strains could be accelerated or automated as described before.

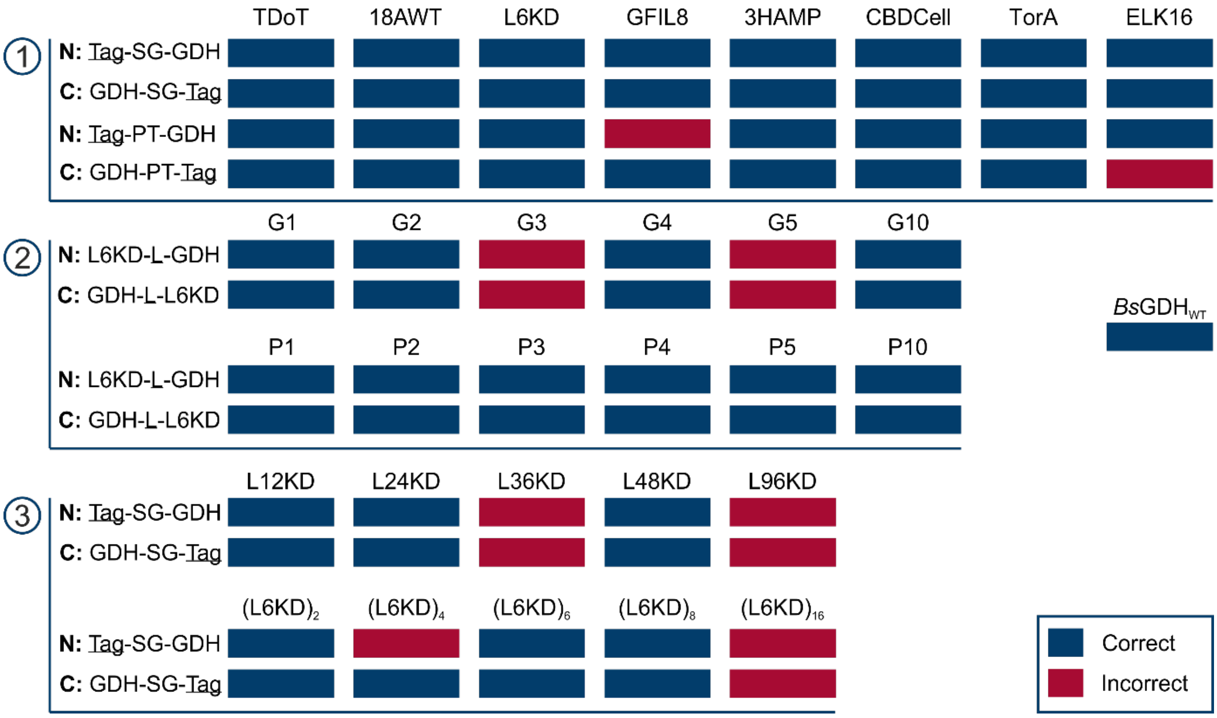


**Figure S1:** Overview of 76 tested *Bs*GDH-CatIB combinations and *Bs*GDH$_{WT}$ control using semi-automated cloning workflow. The blue marked constructs were verified by sequencing. The red marked constructs revealed incorrect sequencing results or no colonies were formed at the end of the cloning process.

### *Validation of the overall CatIB workflow after optimization*

For validation of the workflow with optimized cultivation and purification steps as described in the main manuscript, *E. coli* BL21(DE3) with *Bs*GDH-PT-CBDCell was cultivated in a FlowerPlate with 48 biological replicates. After cultivation, the CatIBs of each well were separately purified in an automated manner (see ***Automated protein production and protein purification*** in the main manuscript) and the automated enzymatic assay was performed. Instead of enzyme inactivation with methanol, the enzymatic assay was measured online with a 20-fold diluted CatIB suspension. The enzymatic assay was performed with 42 biological replicates and 6 purified CatIB samples were analyzed as controls without substrate addition (**Figure S2**).

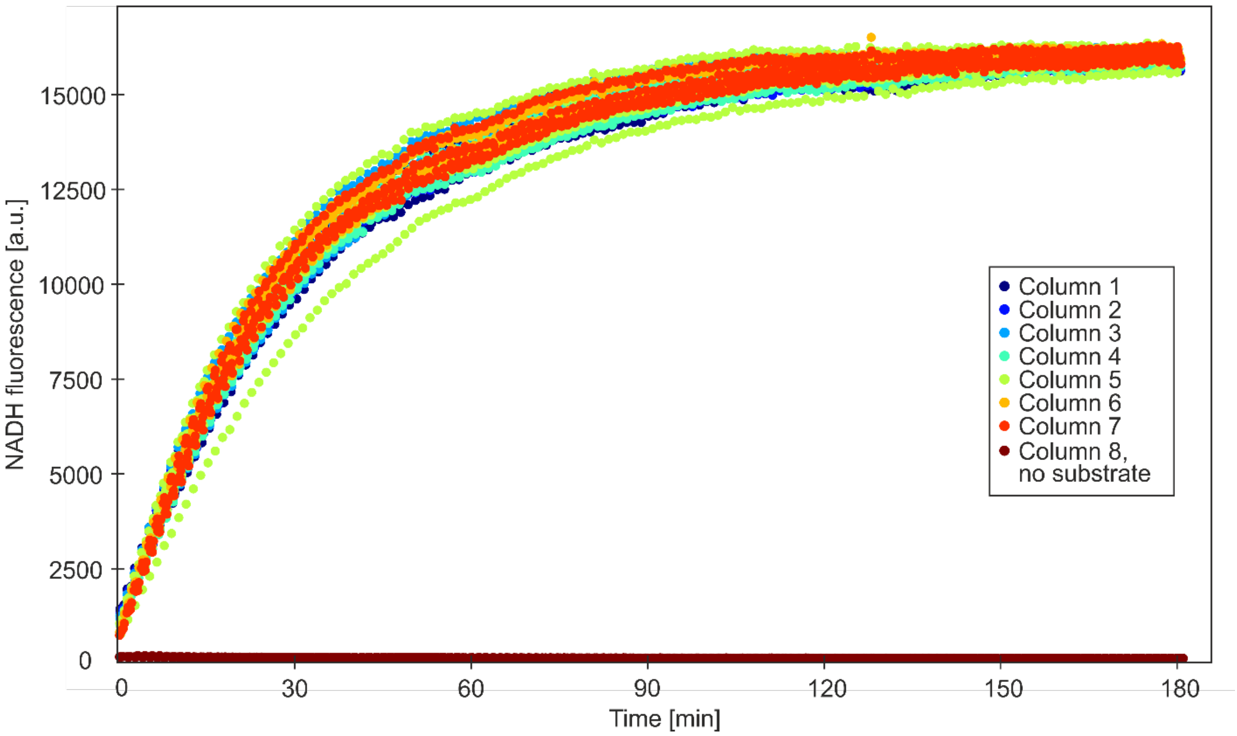


**Figure S2:** NADH fluorescence of *Bs*GDH-PT-CBDCell replicates as a validation study. The CatIBs were purified from 650 µL cells. The enzymatic assay was performed with 40 mM TAE (pH 7), 200 mM glucose and 0.4 mM $NAD^+$. The resuspended and 20-fold diluted CatIBs and the enzyme assay solution were preheated at 40 °C for 10 min before mixing. The final reaction volume was 250 µL. The reaction was performed at 37 °C for 180 min and measured online with an excitation wavelength of 340 nm and an emission wavelength of 470 nm. For testing of the enzyme activity, 42 biological replicates were analyzed, and 6 replicates were used as a negative control without the addition of substrate. One column corresponds to six wells of a FlowerPlate.

The control without substrate did not show a fluorescence signal, clearly indicating that the CatIBs still present in the reaction plate do not interfere with the improved online assay. The 42 replicates with substrate show the expected reaction curve with pseudo-linear behavior in the first 15 min and a saturation curve with comparable final NADH fluorescence values. The relative standard deviation of fluorescence signals after 120 min could be reduced from 11.4 % [1] to only 1.9 % in this improved workflow. At the same time, the online measurement provides higher data resolution as well as fewer pipetting and dilutions steps compared to previous methanol inactivation [1].

### ***Analysis of BsGDH-CatIBs via microscopy, SDS-PAGE and enzymatic assay***

To analyze and compare different *Bs*GDH-CatIBs microscopic analysis (**Figure S3-S7**) and SDS-PAGE (**Figure S7**) were performed.

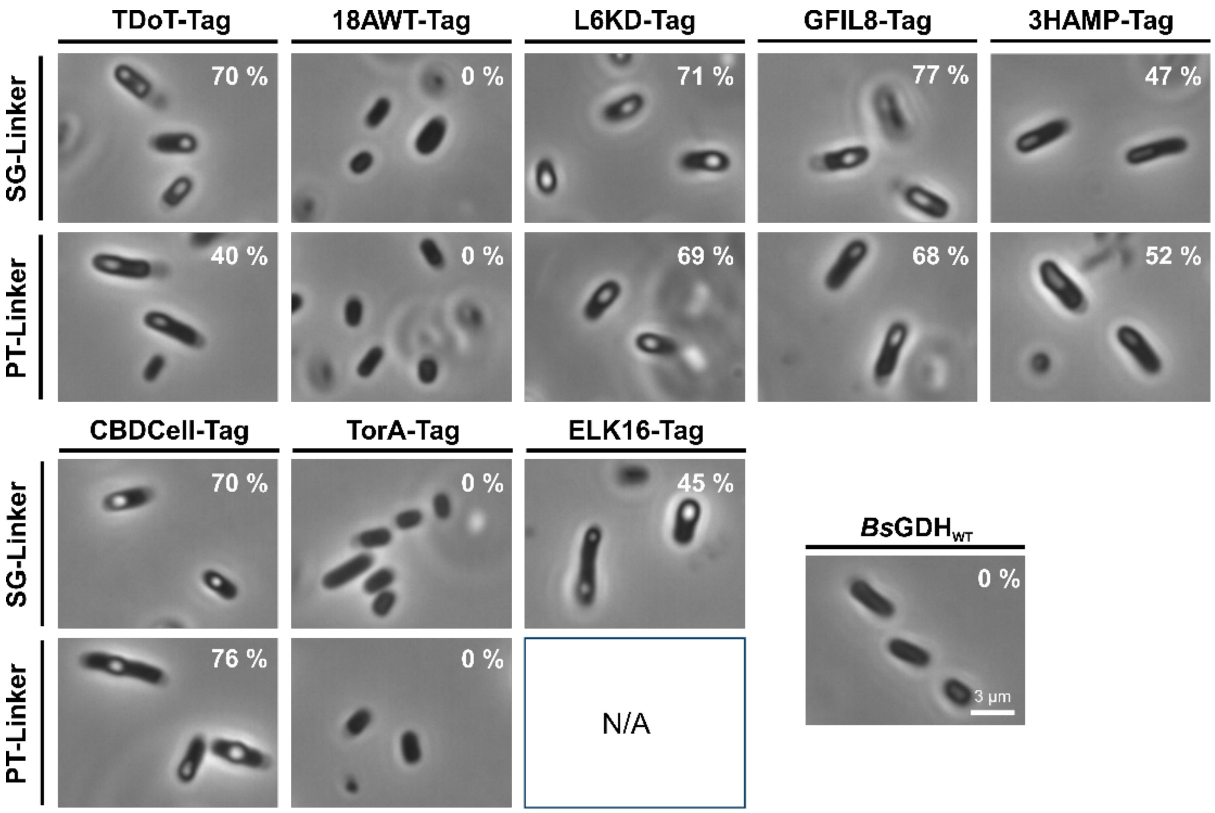


**Figure S3:** Microscopic images of strains producing *Bs*GDH-CatIBs with different linker/aggregation-inducing tag combinations tagged at the C-Terminus of the enzyme and *Bs*GDH$_{WT}$. Percentage of CatIB producing cells for each variant is displayed. Phase contrast microscopy was conducted with a 1000-fold magnification. All strains were cultivated for 72 h at 25 °C in M9 AI medium.

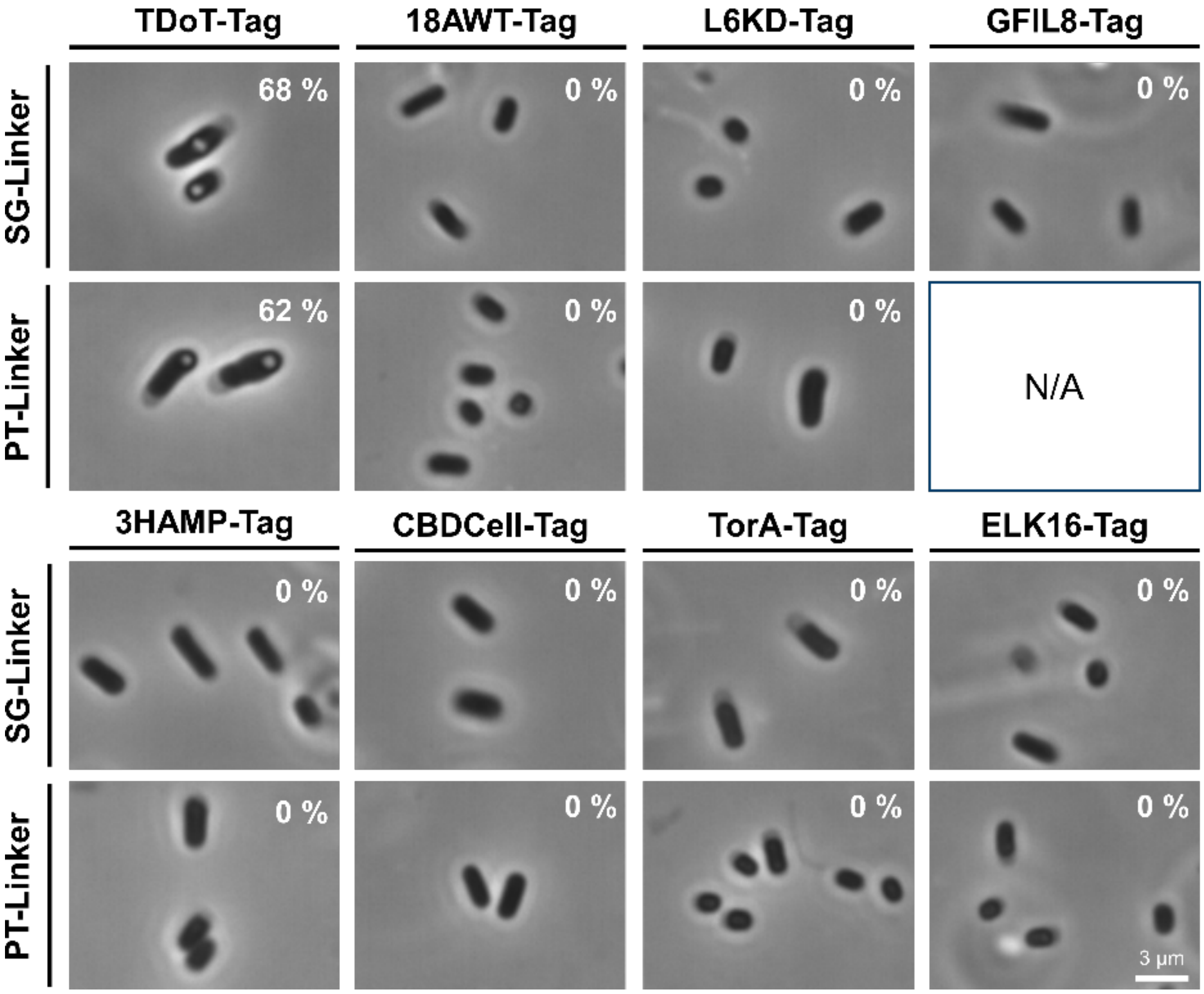


**Figure S4:** Microscopic images of strains producing *Bs*GDH-CatIBs with different linker/aggregation-inducing tag combinations tagged at the N-Terminus of the enzyme. Percentage of CatIB producing cells for each variant is displayed. Phase contrast microscopy was conducted with a 1000-fold magnification. All strains were cultivated for 72 h at 25 °C in M9 AI medium.

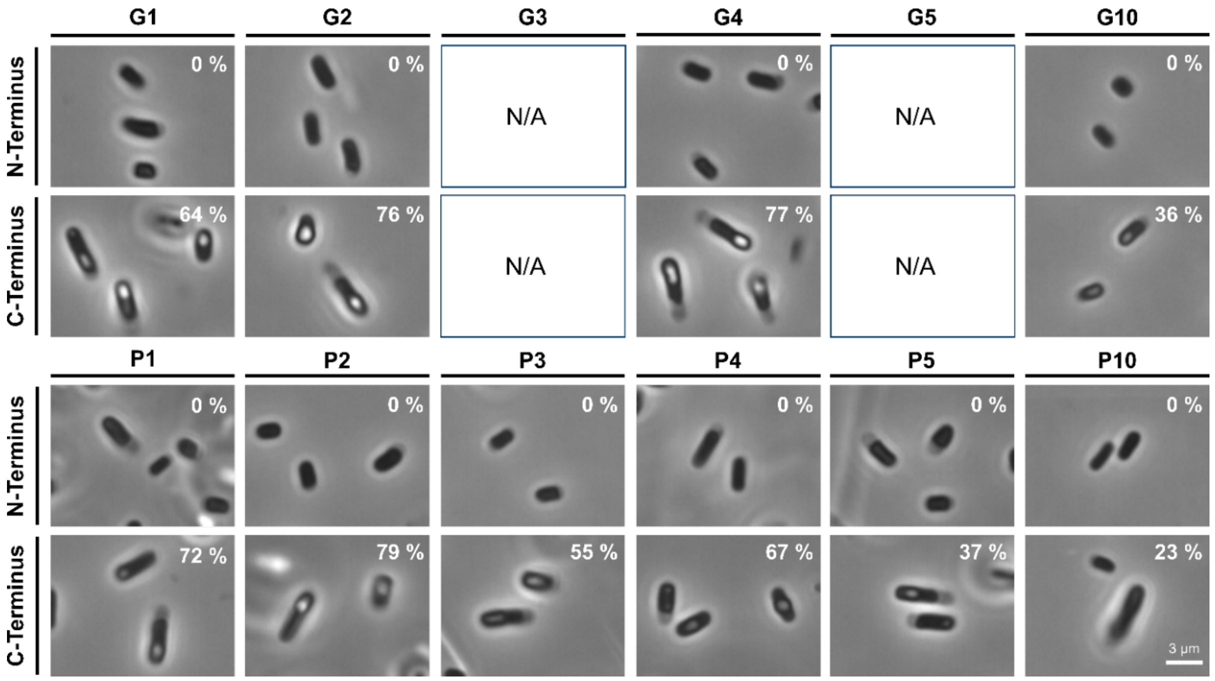


**Figure S5:** Microscopic images of strains producing *Bs*GDH-CatIBs with different lengths of glycine or proline linker tagged at the C- and N-Terminus of the enzyme. Percentage of CatIB producing cells for each variant is displayed. Phase contrast microscopy was conducted with a 1000-fold magnification. All strains were cultivated for 72 h at 25 °C in M9 AI medium.

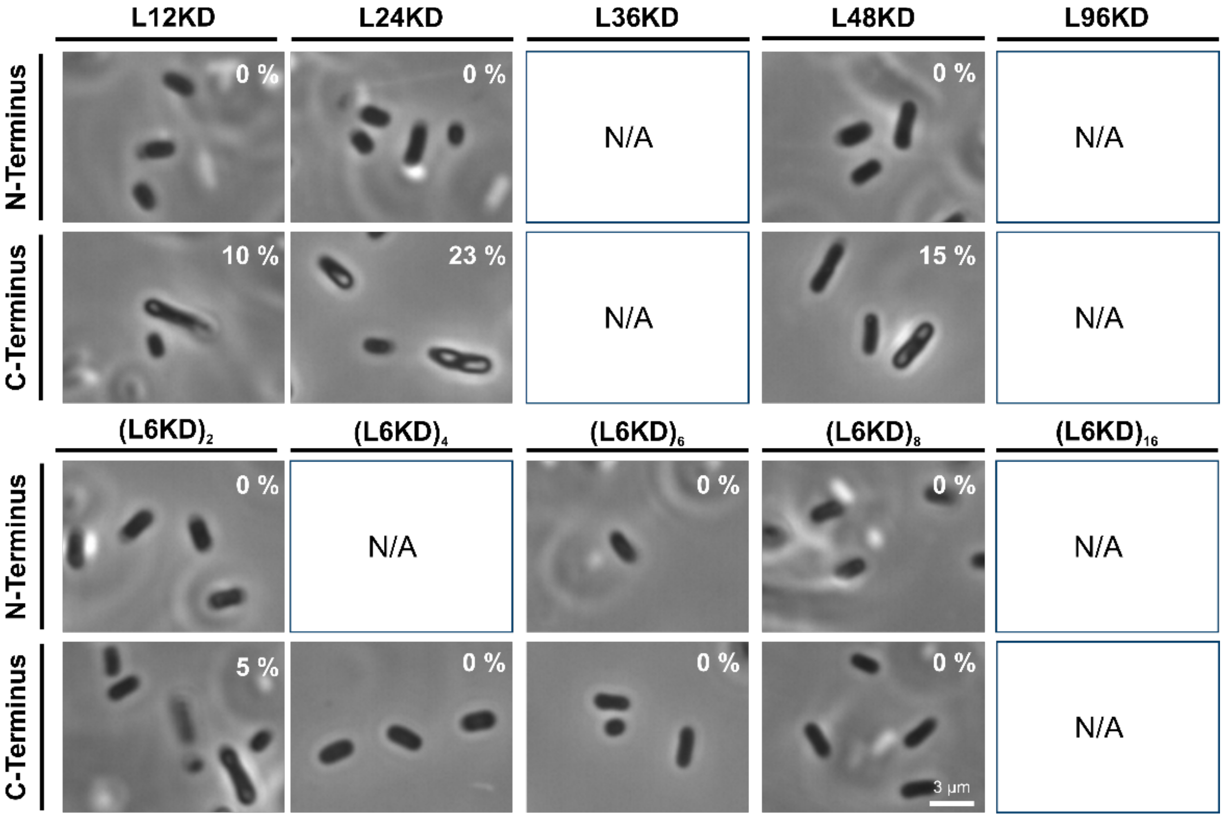


**Figure S6:** Microscopic images of strains producing *Bs*GDH-CatIBs with different lengths of L6KD tag linker tagged at the C- and N-Terminus of the enzyme. Percentage of CatIB producing cells for each variant is displayed. Phase contrast microscopy was conducted with a 1000-fold magnification. All strains were cultivated for 72 h at 25 °C in M9 AI medium.

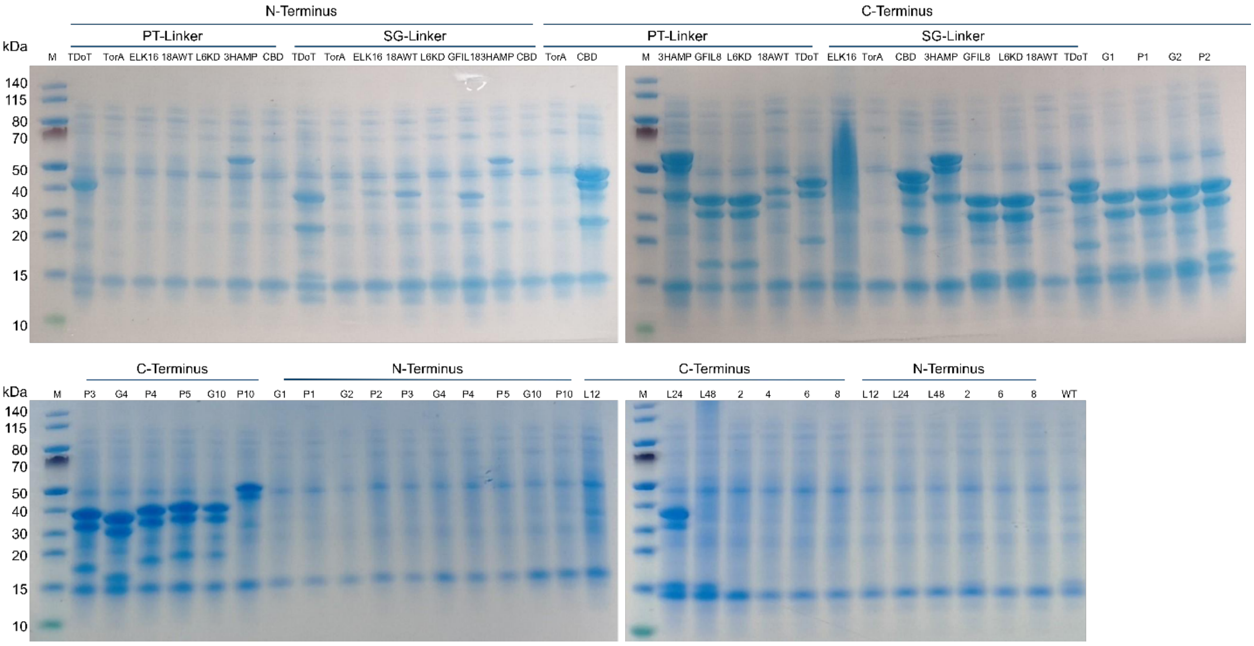


**Figure S7:** Evaluation of 63 *Bs*GDH-CatIB formation and *Bs*$GDH_{WT}$ by SDS-PAGE analysis. After cultivation, the cells were disrupted and the crude cell extract was separated by centrifugation into the soluble protein containing supernatant and the insoluble CatIB-containing pellet fractions. The pellet fraction was washed once with Milli-Q® water. The samples were diluted 1:1 with SDS sample buffer and 15 µL of each sample was loaded onto the gel and stained with SimplyBlue™ SafeStain. The molecular mass of the wildtype *Bs*GDH is 28 kDa. The molecular masses of tags and linkers is listed in **Table S4**.

**Table S4**: Expected molecular weights for linker and tag fragments.

| | | | | | | | | |
|---|---|---|---|---|---|---|---|---|
| **Linker/Tag** | SG | PT | G1 | G2 | G3 | G4 | G5 | G10 |
| **Molecular Weight (kDa)** | 0.9 | 1.7 | 0.7 | 1.1 | 1.4 | 1.7 | 2.1 | 3.7 |
| **Linker/Tag** | P1 | P2 | P3 | P4 | P5 | P10 | TDoT | 18AWT |
| **Molecular Weight (kDa)** | 0.9 | 1.4 | 1.9 | 2.4 | 2.9 | 5.4 | 6.1 | 2.7 |
| **Linker/Tag** | GFIL8 | 3HAMP | CBDCell | TorA | ELK16 | L6KD | L12KD | L24KD |
| **Molecular Weight (kDa)** | 1.2 | 19.0 | 10.7 | 4.5 | 2.3 | 1.3 | 2.0 | 3.3 |
| **Linker/Tag** | L36KD | L48KD | L96KD | $(L6KD)_2$ | $(L6KD)_4$ | $(L6KD)_6$ | $(L6KD)_8$ | $(L6KD)_{16}$ |
| **Molecular Weight (kDa)** | 4.7 | 6.0 | 11.5 | 2.2 | 4.0 | 5.9 | 7.7 | 15.1 |

### *Comparison of cultivation conditions with microscopic analysis*

To further investigate the influence of standard CatIB cultivation conditions (3 h at 37 °C and 69 h at 15 °C) compared to the novel cultivation conditions (72 h at 25 °C), microscopic images were generated using a 1,000x magnification. Cells with successful CatIB production were counted and compared to cells without CatIB formation (**Figure S8).**

| | Cells with CatIBs (%) | | | Cells with CatIBs (%) | |
|---|---|---|---|---|---|
| CatIB variant | **25 °C** | **37 - 15 °C** | CatIB variant | **25 °C** | **37 - 15 °C** |
| TDoT-PT-GDH | 62 | 11 | GDH-G1-L6KD | 64 | 56 |
| TDoT-SG-GDH | 68 | 36 | GDH-P1-L6KD | 72 | 65 |
| GDH-PT-CBDCell | 76 | 52 | GDH-G2-L6KD | 76 | 53 |
| GDH-PT-3HAMP | 52 | 6 | GDH-P2-L6KD | 79 | 36 |
| GDH-PT-GFIL8 | 68 | 75 | GDH-P3-L6KD | 55 | 38 |
| GDH-PT-L6KD | 69 | 63 | GDH-G4-L6KD | 77 | 72 |
| GDH-PT-TDoT | 40 | 13 | GDH-P4-L6KD | 67 | 57 |
| GDH-SG-ELK16 | 45 | 75 | GDH-P5-L6KD | 37 | 22 |
| GDH-SG-TorA | 0 | 32 | GDH-G10-L6KD | 36 | 20 |
| GDH-SG-CBDCell | 70 | 75 | GDH-P10-L6KD | 23 | 0 |
| GDH-SG-3HAMP | 47 | 16 | GDH-SG-L12KD | 10 | 0 |
| GDH-SG-GFIL8 | 77 | 63 | GDH-SG-L24KD | 23 | 86 |
| GDH-SG-L6KD | 71 | 79 | GDH-SG-L48KD | 15 | 71 |
| GDH-SG-TDoT | 70 | 0 | GDH-SG-$(L6KD)_2$ | 5 | 0 |

**Figure S8**: Influence of cultivation temperature on CatIB formation analyzed *via* microscopy. The cultivations were performed at 25 °C (72 h) or 37 °C (3 h) + 15 °C (69 h) with M9 AI medium in a FlowerPlate. Microscopic images of all 63 strains were generated using a 1,000x magnification. The numbers indicate the percentage of CatIB producing cells given the overall number of counted cells.

### *Analysis of volumetric activity for selected BsGDH variants*

Three CatIB variants (*Bs*GDH-PT-3HAMP, *Bs*GDH-PT-CBDCell, *Bs*GDH-PT-18AWT) were tested for their specific volumetric activity $P_v$ at the two different cultivation conditions (shift from 37 °C to 15 °C or constant 25 °C) to test the influence of the temperature on the specific $P_v$ (**Figure S9**). To determine the productivity of the CatIBs, the tested strains were produced in a larger scale in shake flasks. A manual purification and enzyme assay were performed and the CatIB dry weight was determined [2].

The results for all three CatIB variants showed that the cultivation at 25 °C positively influenced the productivity of the CatIBs. Even if the specific $P_v$ of *Bs*GDH-PT-18AWT was lower than 10 g $L^{-1}$ $d^{-1}$ $g_{CatIB}^{-1}$, this variant in general only showed activity after 25 °C cultivation. Moreover, with a 25 °C cultivation, the specific $P_v$ of *Bs*GDH-PT-3HAMP was increased by approx. 40 % and *Bs*GDH-PT-CBDCell was increased by approx. 18 %.

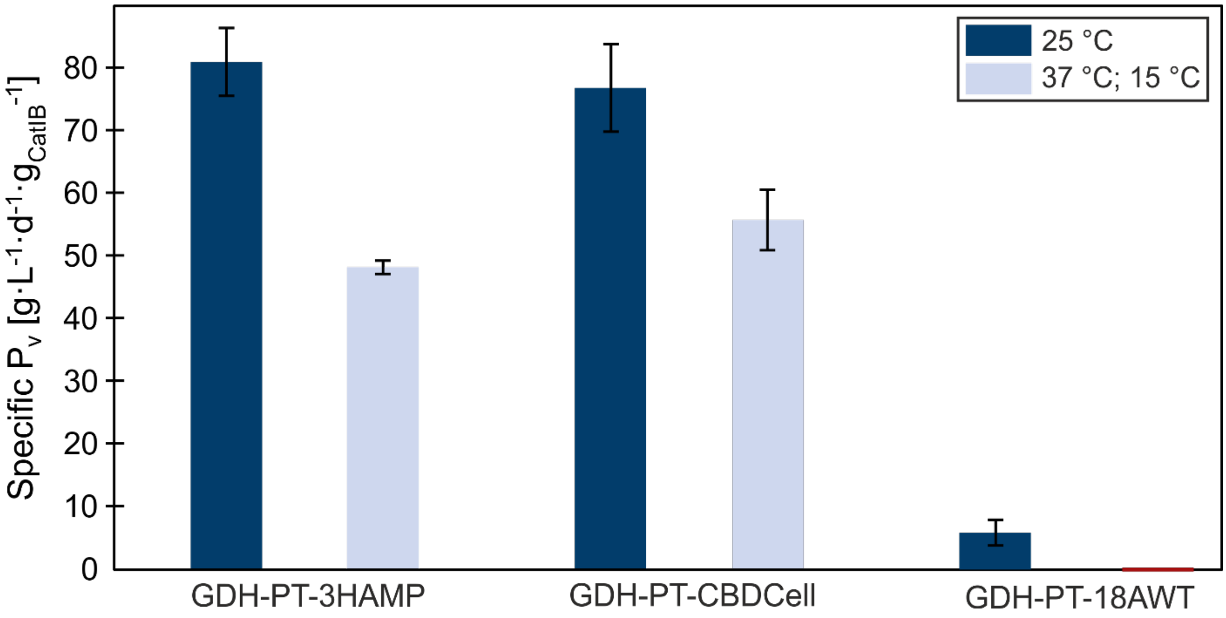


**Figure S9:** Influence of cultivation temperature on specific volumetric productivity of *Bs*GDH-CatIBs. The standard deviation of the technical triplicates for all CatIB variants were calculated.

***Mathematical description of process and calibration model***

According to Bayes' theorem, posterior probabilities can be calculated using prior probabilities and likelihoods. The following equations summarize the Bayesian process model, which is shown as a computation graph in the main manuscript (**Figure 8**):

$$k_{\mathrm{std}} \sim \mathrm{HalfNormal}(\sigma = 0.2)$$

$$k_{\mathrm{mean}} \sim \mathrm{HalfNormal}(\sigma = 0.1)$$

$$t_{\mathrm{offset}} \sim \mathrm{LogNormal}(\mu = \log_{10}(0.283), \sigma = 0.1)$$

$$t_{\mathrm{plate\_to\_reader}} \sim \mathrm{LogNormal}(\mu = \log_{10}(0.5), \sigma = 0.05)$$

$$\mathrm{batch\_effect}(i) \sim \mathrm{LogNormal}(\mu = 0, \sigma = 0.1)$$

$$\mathrm{cf\_nadh\_assay}\ (j) \sim \mathrm{LogNormal}(\mu = 0, \sigma = 0.1)$$

$$S_0 \sim \mathrm{LogNormal}(\mu = \log_{10}(0.2), \sigma = 0.1)$$

$$k_{\mathrm{variant}}(v) = \mathrm{LogNormal}(\mu = \log_{10}(k_{\mathrm{mean}}), \sigma = k_{\mathrm{std}})$$

$$k_{\mathrm{batch}}(i, v) = k_{\mathrm{variant}}(v) \cdot \mathrm{batch\_effect}(i)$$

$$k_{\mathrm{assay}}(i, j, v) = \frac{\mathrm{cf\_nadh}_{\mathrm{assay}}(j) \cdot k_{\mathrm{batch}}(i, v)}{50}$$

$$t(c) = c \cdot t_{\mathrm{offset}} + t_{\mathrm{plate\_to\_reader}}$$

$$Y_{\mathrm{pred}} = P(c, i, j, s) = S_0 \cdot \left(1 - e^{\left(k_{\mathrm{assay}}(i,j,s) \cdot t(c)\right)}\right)$$

$$\mathcal{L}(\theta_{\mathrm{pm}} | Y_{\mathrm{obs}}) = \phi_{\mathrm{cm}}(Y_{\mathrm{obs}}, Y_{\mathrm{pred}})$$

$\mathrm{HalfNormal}$ and $\mathrm{LogNormal}$ indicate the choice of a probability distribution defining the prior belief in the respective parameter. Indices $c, i, j$ and $v$ refer to the column in the assay microtiter plate, the cultivation well, the well in the microtiter plate of the assay, and the CatIB variant, respectively. The prior for $k_{\mathrm{variant}}$ is defined by the mean and the standard deviation of the population, which are expressed as hyperpriors $k_{\mathrm{mean}}$ and $k_{\mathrm{std}}$. Since the reaction is started column-wise by addition of the reaction substrate, a column-specific time offset $t_{\mathrm{offset}}$ is modelled, which was measured experimentally to be around 17 seconds per columns. Similarly, the time to put the plate to the reader was measured as 20 seconds. These measurements were chosen as the mean of the respective prior distributions. $\mathrm{cf\_nadh\_assay}$ reflects the pipetting error during dilution. The batch effect between biological replicates is represented by the respective parameter and is dependent on the cultivation well $i$. In the prediction of the product concentration in a specific well $j$ in the microtiter plate of the assay, the effect of the priors for the variants, the time offset, the pipetting error and the batch effect are combined.

In order to derive posterior distributions for all mentioned parameters of the process model $\theta_{\mathrm{pm}}$ in Markov chain Monte Carlo Sampling, a likelihood function $\mathcal{L}$ needs to be defined. While homoscedastic, normally distributed measurement noise is often assumed if no knowledge exists, the likelihood function can also be calibrated using known concentrations and measurement readouts. Using this calibration data, the relationship between independent and dependent variable can be modeled as a separate function, which we call calibration model. More details and theory on the methodology of process and calibration models can be found in [3].

In this study, we fitted a calibration model $\phi_{\mathrm{cm}}$ that describes the exponential relationship between fluorescence observation $Y_{\mathrm{obs}}$ and predicted concentration $Y_{\mathrm{pred}}$, obtained from the process model. We applied the Python package `calibr8` to fit the model to the calibration data. An analysis plot of the resulting calibration model is given in **Figure S10**.

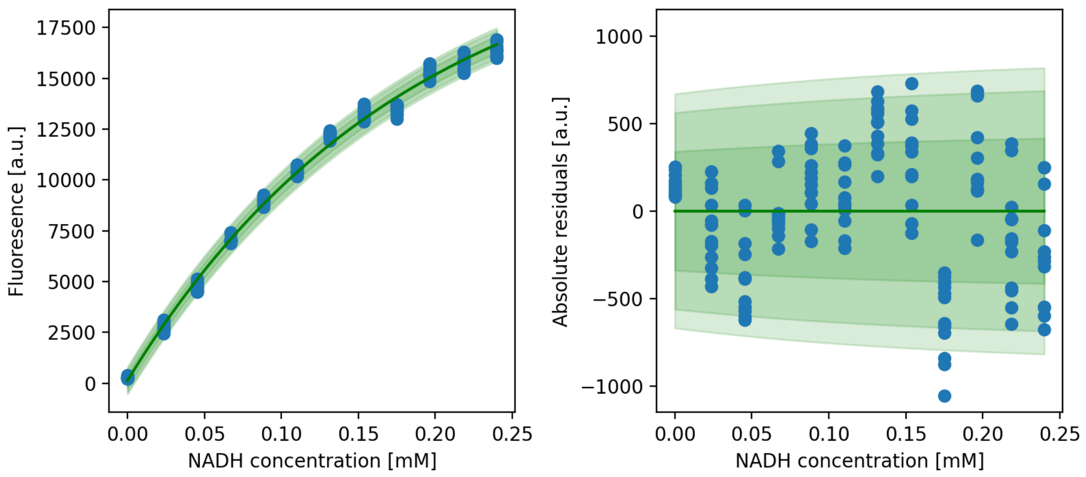


**Figure S10:** Calibration model that describes a normally distributed measurement error for measured NADH fluorescence in the assay. The mean of the normal distribution is described by an exponential trend between true concentration and fluorescence. The standard deviation is linearly increasing with the NADH concentrations, reflecting higher errors for increased NADH concentrations towards the upper detection limit of the plate reader. More details on calibration models can be found in [3].

Essentially, the left panel shows the trend between the independent variable (NADH concentration) and the dependent variable (fluorescence readout). An exponential function was chosen to model this trend, reflecting that the relationship between concentration and readout is non-linear. In addition, the measurement error is increasing with the NADH concentration, which is why a linear function was chosen to describe the width of the likelihood bands in dependency of the NADH concentration. The right panel with the residuals between model and data shows mostly random scattering, with a stronger deviation at around 0.17 mM NADH. Overall, the chosen calibration model describes the data well and was thus applied as a likelihood function in the Bayesian process model. The code to reproduce the calibration model is provided in the accompanying GitHub repository.

## References

[1] K. Küsters, “Accelerated production and screening of catalytically active inclusion body libraries via automated workflows,” RWTH Aachen University, 2022. doi: 10.18154/RWTH-2022-10289.

[2] K. Küsters *et al.*, “Construction and characterization of *Bs*GDH-CatIB variants and application as robust and highly active redox cofactor regeneration module for biocatalysis,” *Microb. Cell Factories*, vol. 21, no. 1, p. 108, Jun. 2022, doi: 10.1186/s12934-022-01816-2.

[3] L. M. Helleckes, M. Osthege, W. Wiechert, E. von Lieres, and M. Oldiges, “Bayesian calibration, process modeling and uncertainty quantification in biotechnology,” *PLoS Comput. Biol.*, vol. 18, no. 3, p. e1009223, 2022, doi: 10.1371/journal.pcbi.1009223.
